# Beyond cytotoxicity: pollutant mixtures elicit unconventional epithelial-fibroblast signaling in a human lung air-liquid interface co-culture model

**DOI:** 10.3389/ftox.2025.1722968

**Published:** 2025-12-18

**Authors:** Justine Fredoc-Louison, Maëva Cherrière, Bastien Rival, Suzanne De Araujo, Sabine François, Samir Dekali

**Affiliations:** 1 Biomedical Research Institute of the Armed Forces (IRBA), EBR Department, Emerging Technological Risks Unit (U.RTE), Brétigny-sur-Orge, France; 2 National Institute for Industrial Environment and Risks (Ineris), MIV Department, Experimental Toxicology and Modelling Unit (TEAM), Verneuil-en-Halatte, France

**Keywords:** air–liquid interface model, human lung epithelial cells, fibroblast activation, pollutant mixtures, aluminum oxide nanoparticles, hydrogen chloride, paracrine signaling, non-cytotoxic response

## Abstract

**Introduction:**

Combined exposures to airborne nanoparticles and acidic gases are plausible during industrial accidents and in military settings involving propellants. Aluminum oxide nanoparticles (Al_2_O_3_ NPs), widely used in industry and present in propellant formulations, together with hydrogen chloride (HCl), a corrosive combustion by-product, are relevant co-pollutants whose joint impact on lung remodeling remains poorly defined. This study aimed to investigate early airway responses to repeated apical exposures to Al_2_O_3_ NPs, HCl, or their mixture in a human air–liquid interface co-culture model of alveolar epithelial (hAELVi) and fibroblast (MRC-5) cells.

**Methods:**

Cells were exposed daily for four days, and epithelial viability, barrier integrity, and mediator release were assessed. Conditioned media from exposed cultures were applied to naïve fibroblasts to evaluate proliferation and migration responses.

**Results:**

Repeated exposures did not induce cytotoxicity, barrier disruption, or increases in canonical pro-fibrotic or pro-inflammatory mediators such as TGF-β1, CTGF, or IL-8. However, conditioned media from exposed epithelial cells consistently triggered fibroblast activation through non-canonical epithelial–mesenchymal signaling pathways. These effects occurred at sub-toxic exposure levels.

**Discussion:**

These findings indicate that early sub-lethal co-exposure to Al_2_O_3_ NPs and HCl can trigger fibroblast activation independently of classical cytokine induction. This unconventional paracrine response suggests a mechanistic link between early epithelial stress and fibroblast-driven remodeling, underscoring the importance of investigating non-canonical pathways in lung responses to mixed environmental pollutants.

## 1 Introduction

Chronic inhalation of airborne pollutants, including engineered nanoparticles (NPs) and acidic gases, is a major environmental and occupational health concern, linked to chronic respiratory diseases, such as idiopathic pulmonary fibrosis (IPF), chronic obstructive pulmonary disease (COPD), and asthma (Bahri and Abidi, 2010; Greenberg et al., 2007). Among these pollutants, aluminum oxide (Al_2_O_3_) nanoparticles (NPs), widely used in industrial processes, have been detected in ambient air and shown to impair lung homeostasis *in vivo* and *in vitro* (Kim et al., 2010; Böhme et al., 2014). Similarly, hydrogen chloride (HCl), frequently released during industrial accidents, combustion events, or military activities, is a potent respiratory irritant capable of disrupting epithelial integrity and triggering inflammation (Colunga Biancatelli et al., 2021). Such combined exposures are highly plausible in real-life contexts such as fires, industrial accidents, and battlefield conditions.

At the alveolar level, epithelial cells not only act as a physical barrier but also orchestrate tissue repair through soluble mediators that activate fibroblasts, the central effectors of fibrosis (Kendall and Feghali-Bostwick, 2014; Plikus et al., 2021). Sustained epithelial injury typically involves canonical cytokines including TGF-β1, CTGF, and IL-8 (Wynn, 2008; Chen et al., 2020).

However, emerging evidence suggests that early, sub-lethal epithelial stress may activate fibroblasts through non-canonical, cytokine-independent mechanisms (Dekali et al., 2022; Sayan and Mossman, 2016). Nanoparticles can alter barrier properties and junctional proteins without overt cytotoxicity (Leibrock et al., 2019), and acidic gases like HCl can modify gene expression and permeability at non-lethal doses (Ohwada et al., 2003). Such subtle alterations may transmit signals through extracellular vesicles (EVs), metabolites, or damage-associated molecular patterns (DAMPs; which act as endogenous danger signals), raising the possibility that early “silent” responses to pollutant mixtures initiate remodeling before overt inflammation becomes detectable.

Although the individual effects of engineered NPs and acidic gases on lung cells have been studied, their combined impact remains poorly understood, despite its critical relevance for risk assessment in occupational and accidental contexts where mixed exposures are common (Colunga Biancatelli et al., 2021; Sayan and Mossman, 2016; Bourgois et al., 2019). A central unresolved question is whether such exposures trigger additive, synergistic, or unconventional paracrine responses in epithelial-mesenchymal communication.

Despite these insights, most studies have been performed in submerged systems, which underestimate aerosol-induced paracrine signaling. Air-liquid interface (ALI) models preserve epithelial polarization and allow direct aerosol delivery, providing a more realistic platform to investigate early fibrogenic events (Sengupta et al., 2022; Srinivasan et al., 2015; Clippinger et al., 2016).

While ALI models represent a clear improvement over submerged systems by enabling apical exposures and epithelial polarization (Upadhyay and Palmberg, 2018), most still rely on epithelial monocultures and fail to account for the role of stromal components. Fibroblasts, in particular, are critical mediators of tissue remodeling, yet are often excluded from *in vitro* studies of inhaled toxicants (Clippinger et al., 2016). To overcome these limitations, recent developments have focused on co-culture systems, organoids, and lung-on-chip platforms that better replicate the multicellular architecture and functional complexity of the human lung (Sengupta et al., 2022; Huh et al., 2010; Purev et al., 2024). In our previous work (Bourgois et al., 2019), the effects of Al_2_O_3_ NPs and HCl were examined in alveolar epithelial monocultures, revealing direct impacts on barrier integrity and cytokine signaling. However, such an approach did not allow investigation of paracrine communication with stromal cells. The present study therefore extends these findings by implementing an ALI co-culture model combining epithelial and fibroblast cells, designed to capture epithelial–mesenchymal interactions and to reveal non-canonical paracrine mechanisms of fibroblast activation. Our ALI co-culture model builds on these advances by combining epithelial and fibroblast cell types under physiologically relevant conditions, thus enabling the investigation of early epithelial-mesenchymal interactions triggered by aerosolized pollutants.

This study specifically focused on sub-lethal exposures, which are highly relevant for regulatory toxicology since real-world pollutant levels rarely cause acute epithelial damage but may initiate long-term remodeling. Identifying early biomarkers of such “silent” responses is essential to improve hazard identification frameworks.

Here, we used a human air-liquid interface co-culture of alveolar epithelial (hAELVi) and lung fibroblast (MRC-5) cell lines to test whether repeated, sub-lethal exposures to Al_2_O_3_ NPs, HCl, or their mixture elicit fibroblast activation through non-canonical pathways (Sengupta et al., 2022; Srinivasan et al., 2015; Clippinger et al., 2016; Upadhyay and Palmberg, 2018; Huh et al., 2010; Purev et al., 2024). Based on prior observations that such exposures can alter epithelial behavior without overt cytotoxicity or classical cytokine induction (Dekali et al., 2022; Leibrock et al., 2019; Ohwada et al., 2003), we hypothesized that pollutant mixtures generate atypical epithelial-derived signals independent of the TGF-β1/CTGF axis. We designed the study to quantify early paracrine effects on fibroblast migration and proliferation while monitoring canonical mediators and barrier integrity.

Building on this rationale, our work provides the first evidence that combined sub-lethal exposures to Al_2_O_3_ NPs and HCl can trigger early fibroblast activation through unconventional epithelial-mesenchymal communication in a human alveolar co-culture model under air–liquid interface conditions.

## 2 Materials and methods

### 2.1 Physico-chemical characterization of alumina nanoparticles suspensions and mixtures with hydrochloric acid

Alumina nanoparticles (Al_2_O_3_ NPs) were provided by Sigma-Aldrich (#718475, St. Louis, MO, United States) with the following physico-chemical specifications: primary size 13 nm, crystalline phase gamma/delta (γ/δ), specific surface area (85–115 m^2^/g) experimentally confirmed by the Brunauer, Emmet, and Teller method (BET) (97 m^2^/g), pH (4.5–5.5), and purity 99.8%. Hydrochloric acid (HCl) was also purchased at Sigma-Aldrich (#258148, St. Louis, MO, United States).

Al_2_O_3_ NPs were extemporaneously suspended in sterile water (#B230521, Fresenius, Germany) with or without HCl according to a standardized and sequential protocol: vortex for 1 min, ultrasonic bath for 1 min and vortex for 1 min. Final concentrations were respectively 1 mg/mL and 1.37 mM prior to cell exposure.

To prevent agglomeration and ensure reproducible nebulization, nanoparticle suspensions were briefly sonicated (3 min, 40 W). The sonication parameters were optimized in preliminary tests to achieve stable hydrodynamic size distributions without affecting particle crystallinity or inducing detectable aluminum dissolution, as confirmed by DLS and ICP-OES analyses.

The following parameters were then studied: morphology of the NPs and their aggregates/agglomerates, size distribution, zeta potential and pH. To study the morphology of the NPs and their aggregates/agglomerates, the suspensions of NPs were deposited on adhesive supports then incubated in an oven for 5 min at 37 °C. The samples were then placed immediately into the chamber of the Quanta 200 scanning electron microscope for image acquisition. The size distribution and zeta potential measurements were carried out at 25 °C by the “Dynamic Light Scattering” (DLS) method using a ZetaSizer Ultra (Malvern, United Kingdom). The analyzes were performed with the ZS Xplorer software suite (Malvern, United Kingdom). The pH measurements of the different suspensions of nanoparticles were carried out at room temperature (RT) with an S220 Seven-Compact pH/ion pH meter (Mettler Toledo, Switzerland).

### 2.2 Aerosol dosimetry using quartz crystal microbalance (QCM)

Aerosol deposition was quantified using a quartz crystal microbalance (Vitrocell® sQCM 12,Vitrocell Systems Germany) integrated into the CLOUD-12 exposure module. Prior to exposure, the Aeroneb® Pro nebulizer (Aerogen Ltd., Ireland) was calibrated using 200 µL of sterile PBS (without CaCl_2_ and MgCl_2_) to verify output rate, which was considered acceptable between 0.2 and 0.8 mL/min. For dosimetry, test suspensions were nebulized directly into the exposure chamber using the following conditions: sterile water, HCl (1.37 mM), Al_2_O_3_ NPs (1 mg/mL), and combination of Al_2_O_3_ NPs and HCl. Three replicate nebulizations (200 µL each) were performed per condition. The concentration of HCl (1.37 mM, corresponding to ∼50 ppm) was selected to mimic acute occupational or accidental exposure scenarios, representing a sub-lethal but biologically relevant insult able to trigger epithelial stress without causing massive cytotoxicity. The sQCM 12 was successively positioned in three different locations at the bottom of the exposure module, and the deposited mass were recorded in real time. Between each exposure condition, the exposure chamber was cleaned with 70% ethanol, and the Aeroneb® was rinsed twice with 500 µL sterile water under a laminar flow hood to prevent cross-contamination. This procedure enabled reproducible quantification of deposited mass under air-liquid interface (ALI) exposure conditions.

### 2.3 Cell culture

To establish our model, 2 cell lines were used: hAELVi alveolar epithelial cells (#INS-CI-1015, InSCREENex, Germany) and MRC-5 fibroblasts (#CCL-171, ATCC, United States). hAELVi cells were cultured in huAEC medium (#INS-ME-1013, InSCREENex, Germany). MRC-5 cells were cultured in cell medium Minimum Essential Medium (#30–2003, ATCC, United States) supplemented with 10% (v/v) fetal calf serum (#F7524, Sigma-Aldrich, St. Louis, MO, United States) and 1% (v/v) penicillin/streptomycin (#15140-122, Gibco, United States). Both cell lines were cultured in T75 flasks at 37 °C, 5% CO_2_ and 95% humidity. The medium was changed every two to 3 days and cells were passaged at 90% of confluence.

### 2.4 Co-cultures and transepithelial electrical resistance (TEER) follow-up

One day before seeding, the apical and basolateral compartments of Transwell® (polyester, 1.12 cm^2^, 0.4 μm pores) (Corning, United States) were coated with huAEC Coating Solution (#INS-SU-1018, InSCREENex, Germany). The day of seeding, the Transwell® were rinsed with PBS buffer (#10010-015, Gibco, United States).

MRC-5 fibroblasts were incubated on the basal side of the Transwell® (2.5 × 10^4^ cells/cm^2^) and allowed to attach for 3 h at 37 °C, 5% CO_2_ and 95% humidity. After incubation the Transwell® were placed into huAEC medium-containing wells of a 12-well plate and hAELVi cells (2.5 × 10^5^ cells/cm^2^) were seeded in the apical compartment. The selected seeding ratio (1:10, fibroblasts to epithelial cells) was empirically determined in preliminary optimization experiments in order to obtain a stable and reproducible epithelial barrier, as reflected by consistent TEER measurements, while maintaining fibroblast viability in the basal compartment. This ratio was therefore considered appropriate to mimic the epithelial predominance in the distal alveolar region while preserving epithelial-mesenchymal crosstalk.

The day the bi-cultures were seeded (with hAELVi and MRC-5 cells) was considered as day 0. On day 3, the culture medium was aspirated from the apical compartment to allow cells to be cultured at the air-liquid interface (ALI). The culture medium was then changed every 2 days in the basolateral compartment. In parallel, monocultures with either hAELVi or MRC-5 cells were prepared following the same conditions.

Every 2 days, from day 3 to day 28 post-seeding, measurements were performed on cell mono- and co-cultures using an EVOM Manual instrument (WPI) equipped with an Endohm® 12 chamber. As the cells had been cultured at air-liquid interface, it was necessary to incubate them with huAEC culture medium at 37 °C for 1 h in the apical compartment to equilibrate them before reading the TEER values (expressed in Ohm). Endohm® 12 chamber was pre-filled with pre-warmed (37 °C) hUAEC cell medium. Then, Transwell® membranes were placed into the chamber and resistance measured using the EVOM Manual. Blank controls, consisting of coated filter membranes without cells were measured. TEER was calculated with the following formula:

TEER (Ω.cm^2^) = (measured TEER–blank insert value) x Transwell area (1.12 cm^2^).

To better characterize the temporal dynamics of epithelial barrier formation, TEER values obtained from day 3 to day 28 were further modeled using a double sigmoidal nonlinear regression, as previously described by Brookes et al. (2021). This model enables the identification of two sequential phases of barrier development and is particularly suited for describing biphasic TEER kinetics observed in ALI cultures. The fitted equation includes six parameters: the maximum TEER value, the slopes (steepness) and inflection points of each phase.

TEER values were fitted using a double-sigmoidal regression model applied to each independent biological replicate. The extracted parameters (baseline, maximal resistance, and inflection time) were averaged across replicates for statistical comparisons.

Goodness-of-fit was assessed using adjusted R^2^, RMSE (root mean square error), and standard error of the estimate. Residuals were evaluated for normality to confirm the robustness of the fit. This approach allowed comparative analysis of TEER kinetics across monoculture and co-culture conditions, and helped validate the temporal maturation of the epithelial barrier.

### 2.5 Immunolabeling of intercellular junctions

Intercellular junctions play a crucial role in epithelial barrier function, therefore cultures were stained for the tight-junction proteins ZO-1 and Occludin as well as the adherent junction protein E-Cadherin. At day 17, cells were rinsed in pre-warmed (37 °C) HBSS (with CaCl_2_ and MgCl_2_) (#14025-050, Life Technologies, United States) and fixed for 15 min at RT in 4% (v/v) paraformaldehyde (#119690010, Thermo Fisher Scientific, United States). Fixed cells were permeabilized for 3 min with 0.5% (v/v) Triton X100 (#X100-500 ML, Sigma-Aldrich, St. Louis, MO, United States) at RT. Aspecific sites were blocked with HBSS buffer (with CaCl_2_ and MgCl_2_) containing 2% bovine serum albumin (#A7030-100G, Sigma-Aldrich, St. Louis, MO, USA) for 1 h at RT. Anti-ZO1 (dilution 1:100, #40–2300, ThermoFisher Scientific United States), anti-occludin (dilution 1:500, #40–4700, Thermo Fisher Scientific, United States) or anti-E cadherin (dilution 1:2000, #13–1700, Thermo Fisher Scientific, United States) were incubated for 1 h with hAELVi cells in the apical compartment. After washing the inserts with HBSS the corresponding secondary antibodies, Alexa Fluor 488 (dilution 1:2000, #A32731, Thermo Fischer Scientific, United States) or Alexa Fluor 555 (dilution 1:2000, #A32727, Thermo Fisher Scientific, United States), were incubated with hAELVi cells for 1 h at RT. Cells were stained with Hoechst 33342 (#H3570, Thermo Fisher Scientific, United States) to visualize nuclear DNA. Transwell® membranes were carefully cut, then mounted on a SuperFrost + slide with ProLong™ Diamond Antifade Mountant (#P36970, Thermo Fisher Scientific, United States).

### 2.6 Air-liquid interface exposures to Al_2_O_3_ NPs/HCl mixtures and submerged exposures to positive controls (LPS, bleomycin)

To expose the cells at ALI, the Vitrocell® CLOUD 12 (Vitrocell System, Germany) was used. This device nebulizes a liquid suspension into the apical compartment of the bicameral chambers, in a thermostatically controlled chamber. To mimic repeated exposure to pollutants nebulizations were performed once a day from day 17 to day 20 at ALI. Cell monocultures (hAELVi and MRC-5) and cell co-cultures (hAELVi/MRC-5) were exposed by nebulization to one of the following four conditions:

1. Sterile water (#B230521, Fresenius, Germany) corresponding to control cells;
2. Al_2_O_3_ NPs suspended in sterile water at 1 mg/mL (#718475, Sigma-Aldrich, St. Louis, MO, United States);
3. HCl at 1.37 mM (#258148, Sigma-Aldrich, St. Louis, MO, United States);
4. Mixtures of Al_2_O_3_ NPs (1 mg/mL) with HCl (at 1.37 mM).

Prior to each exposure, particles were resuspended extemporaneously as described in Section 2.1. Each liquid suspension was nebulized for 40 s, followed by a deposition time of 5 min. After each exposure, the cells were returned to their companion plate in the incubator at 37 °C.

In parallel, two positive control conditions were performed under submerged exposure to respectively validate epithelial responsiveness to pro-inflammatory and pro-fibrotic stimuli:

- Lipopolysaccharide (LPS, 1 μg/mL, from *E. coli* O111:B4, #L2630, Sigma-Aldrich, United States) for 96 h;
- Bleomycin sulfate (0.15 μg/mL, #B1141000, Sigma-Aldrich, United States) for 96 h.

These submerged positive control exposures were performed at day 17 post-seeding on cells, in parallel with ALI exposure experiments.

Quantification by ICP-OES confirmed that the total cumulative deposition of Al_2_O_3_ NPs after the 4-day repeated exposure protocol corresponded to ∼1.1 μg/cm^2^. This value reflects the sum of four daily nebulizations and was consistent across independent experiments, ensuring that the reported dose refers to the cumulative deposited mass.

Immediately after ALI exposure, apical rinses were collected and analyzed for chloride anions according to NF EN ISO 10304 (2009). Results were expressed in µg/insert and converted to µg/cm^2^ (Transwell® area 1.12 cm^2^) and nmol H^+^/cm^2^ using 1:1 stoichiometry (HCl → H^+^ + Cl^−^; 35.45 µg Cl^−^ = 1 µmol). The limit of quantification (LQ) was 0.15 µg/insert (and 0.10 µg for basal medium); values below these thresholds were treated as non-quantifiable. Formulas used: Dose (Cl^−^, µg/cm^2^) = (µg/insert)/1.12; Dose (H^+^, nmol/cm^2^) = (µg/cm^2^) × 1000/35.45. For reference, the LQ corresponds to 0.134 μg/cm^2^ and 3.78 nmol H^+^/cm^2^ on a 1.12 cm^2^ insert.

### 2.7 Prestoblue® cell viability assay

On day 21, i.e. 24 h after the final exposure of the 4-day repeated exposure protocol, the PrestoBlue® assay (#A13261, Life Technologies, United States) was performed on mono-cultures and on co-cultures as previously described by Dekali et al. (2014).

The medium was discarded and both the apical and basolateral compartments were washed twice with pre-warmed (37 °C) HBSS (with CaCl_2_ and MgCl_2_). Then a solution of Prestoblue® diluted 10-fold in huAEC culture medium was incubated with cells in the apical and basolateral compartments in the dark in a 37 °C incubator with an atmosphere of 5% CO_2_ and 95% humidity. After 1 h of, the apical and basolateral media were collected in a 96-well plate and the cell viability was estimated by measuring the emitted fluorescence with a cyto-fluorometer (λexcitation = 560 nm, λemission = 590 nm. The percentage of cell viability for each sample was calculated compared to non-exposed cells (corresponding to 100% viability).

### 2.8 Quantification of canonical and non-canonical pro-fibrotic and inflammatory mediators

To assess the inflammatory and pro-fibrotic response, inflammatory cytokine IL-8, Collagen Type I, growth factors CTGF and TGF-β1 were measured in the basolateral medium, as the apical surface is air-exposed under ALI conditions and does not contain recoverable fluid, using an enzyme-linked immunosorbent assay (ELISA). Following the repeated daily exposures, basolateral medium was collected 24 h after the final exposure, i.e., at 96 h after the start of the exposure protocol, a time point selected to capture early paracrine signals before secondary remodeling or degradation processes could occur. Basolateral media were collected separately from hAELVi monocultures, MRC-5 monocultures, and hAELVi/MRC-5 co-cultures, exposed under each condition (sterile water, Al_2_O_3_ NPs, HCl, or Al_2_O_3_ NPs + HCl). For each experimental condition, three independent biological replicates were performed (n = 3). The collected media were centrifuged at 14,000 rpm at 4 °C for 10 min to remove debris and nanoparticles, aliquoted, and stored at −80 °C until ELISA analyses. No pooling of samples was performed. Total protein content was quantified by BiCinchoninic acid Assay for each sample individually to normalize cytokine and growth factor concentrations. Prior to the assay, total protein concentration was measured using a colorimetric assay (#23225, Thermo Fisher Scientific, USA) as recommended by the supplier. This normalization accounted for differences in cell number and biomass between monoculture and co-culture conditions, ensuring that measured concentrations reflect secretion per unit cellular material. Re-analysis of non-normalized ELISA concentrations confirmed the same qualitative pattern, indicating that normalization did not change the interpretation of the results. ELISA assays for CTGF (#ab261851, Abcam, United Kingdom), TGF-β1 (#DB100C, Bio-Techne, USA), Collagen Type I (ab285250, Abcam, United Kingdom) and IL-8 (#DB800C, Bio-Techne, USA) were performed according to the protocols provided in the kits. Concentration of protein of interest (expressed in pg/mL) was related to the amount of total protein (expressed in µg). For each analyte, concentrations are therefore expressed in pg/µg of total protein.

To broaden the inflammatory profiling, a semi-quantitative analysis of 105 soluble proteins was performed using the Human XL Cytokine Array Kit (ARY022B, R&D Systems, USA) on pooled basolateral media (n = 3 independents experiments) from hAELVi, MRC-5 and hAELVi/MRC-5 cultures exposed to each condition (sterile water, Al_2_O_3_ NPs, HCl, or Al_2_O_3_ NPs + HCl). Membranes pre-coated with capture antibodies were incubated overnight at 4 °C with 1.5 mL of sample in Array Buffer 6, followed by biotinylated detection antibody cocktail, streptavidin-HRP, and chemiluminescent substrate. Finally, the chemiluminescent mixture was added to each membrane before reading with the device ChemiDoc XRS + System (BioRad, USA). The signal produced is proportional to the amount of bound analyte. Data were finally analyzed using Fiji image processing software (Schindelin et al., 2012). The analysis module used was the “Protein array analyzer” as described by (Watanabe et al., 2005).

### 2.9 Follow-up of cell adhesion, proliferation and migration using xCELLigence real-time cell analysis (RTCA)

Real time monitoring of MRC-5 living cells was performed using a xCELLigence RTCA DP (dual-purpose) instrument (Agilent, USA), placed in an incubator at 37 °C, 95% humidity and 5% CO2. The xCELLigence system measures electrical impedance, which is reported as a dimensionless parameter called Cell Index (CI), reflecting changes in cell adhesion, proliferation, viability, morphology, or migration.

For cell adhesion and proliferation, measurements were carried-out using 16-well E-plates (#300600890, Agilent, USA). Initially, 100 µL of huAEC cell culture medium (#INS-ME-1013, InSCREENex, Germany) was added per well for background impedance measurement step. Subsequently, 100 µL of MRC-5 cells (2 × 10^5^ viable cells/well) were added, and allowed to settle in the cell culture hood for 30 min at RT. This incubation time is necessary to allow cells to settle in an evenly distributed pattern at the bottom of the wells. CI measurements were acquired every 10 min over a 24-h period to monitor initial adhesion and proliferation. Subsequently, cells were treated with conditioned media collected from basolateral compartment of hAELVi, MRC-5, or hAELVi/MRC-5 cultures previously exposed at the air-liquid interface (ALI) to sterile water (vehicle control), Al_2_O_3_ NPs, HCl, or a combination of Al_2_O_3_ NPs and HCl (exposure protocols detailed in Section 2.6). Each condition was performed in duplicate wells, and signal acquisition continued every 10 min for an additional 53 h. For xCELLigence experiments, control MRC-5 cells were seeded directly in E-Plate 16 wells, which do not involve apical/basolateral compartments. Results were normalized to the Cell Index measured immediately before treatment application expressed using baseline normalized cell index (BNCI) as described by Bourgois et al. (2019). The BNCI corresponds to the impedance-derived Cell Index normalized to the baseline measurement prior to exposure, allowing comparison across conditions. BNCI is calculated by RTCA Software as the Cell Index at a given time point divided by Cell Index at the selected normalization time point. To compare proliferation kinetics across conditions, the BNCI values over the 53-h period were fitted using a second-degree polynomial curve. The mean proliferation velocity was then calculated as the average slope (i.e., first derivative) of the fitted curve over the 53 h. This method accounts for both linear and nonlinear patterns of cell growth. Standard deviations were estimated from the uncertainty in the regression coefficients, using error propagation formulas.

For migration assays, 16-well CIM-plates were used (#5665817001, Agilent, United States). CIM-plates have upper and lower chambers for each well separated by a microporous membrane (in polyethylene terephthalate) with pore size of 8 µm and in contact with microelectrodes. Prior to exposure, the basolateral chambers were filled with 160 µL of fresh huAEC medium to ensure appropriate nutrient supply during the aerosol exposure. CIM-plates were then assembled under the sterile conditions using CIMPlate 16 assembly tool. Subsequently, 25 µL/well of huAEC cell culture medium were added in upper chambers to cover the membrane surface, and CIM-Plates were incubated for 1 hour at 37 °C, 95% humidity and 5% CO_2_. A background impedance measurement step was then recorded. Subsequently, 100 µL of MRC-5 cell suspension (2 × 10^4^ viable cells/well) were then added in upper chambers. Each condition was performed in duplicates with a programmed signal detection schedule every 10 min for 53 h. Control cells were only incubated with huAEC cell culture. The migration dynamics were expressed as Delta Cell Index (ΔCI), defined as the increase in impedance due to fibroblast migration through the membrane. ΔCI values were analyzed using the same polynomial modeling approach as for proliferation. The mean migration velocity was calculated as the average slope of the fitted curve over 53 h. Standard deviations were computed similarly, using the regression output.

### 2.10 Statistical analysis

All statistical analyses were performed using GraphPad Prism version 9.5.1 (GraphPad Software, San Diego, CA, United States). Data are expressed as mean ± standard deviation (SD) from at least three independent experiments, with the exact number of replicates (n) specified in each figure legend. Individual values are plotted to illustrate variability. Normality of data distribution was assessed using the Shapiro–Wilk test.

For comparison of means between exposure groups and the control (sterile water), one-way ANOVA followed by Dunnett’s multiple comparisons test was used for normally distributed data. When normality was not met, the Kruskal–Wallis test followed by Dunn’s *post hoc* test was applied. A *p-*value <0.05 was considered statistically significant, and the following notation was used: *p* < 0.05 (*), *p* < 0.01 (**), *p* < 0.001 (***), *p* < 0.0001 (****), ns: not significant.

Curve fitting, regression modeling, and velocity calculations for real-time proliferation and migration data, as well as TEER kinetics, are detailed in Sections 2.4 and 2.9. All graphs and statistical tests were generated using GraphPad Prism. Curve fitting and regression diagnostics were performed using built-in non-linear regression tools with least-squares minimization.

## 3 Results

### 3.1 Validation of epithelial barrier properties in the hAELVi/MRC-5 co-culture model

**FIGURE 1:**
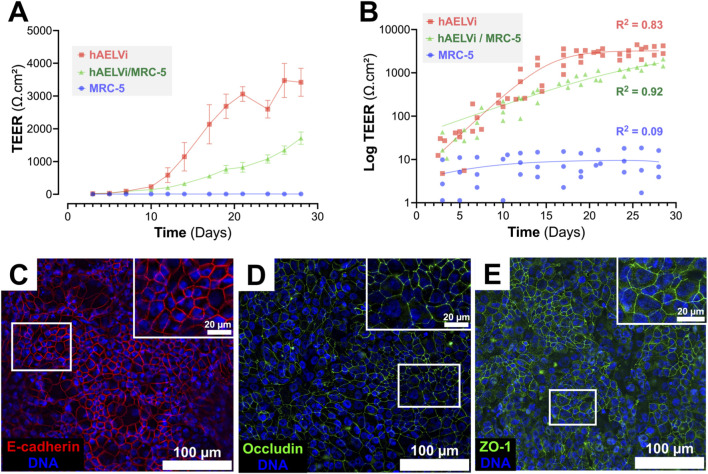
Establishment and validation of the hAELVi/MRC-5 co-culture model at the air-liquid interface (ALI). **(A)** Time-course evolution of transepithelial electrical resistance (TEER) in hAELVi monoculture (red), hAELVi/MRC-5 co-culture (green), and MRC-5 monoculture (blue), cultured on Transwell® inserts under ALI conditions. Data are presented as mean ± SEM (n = 3 for MRC-5 and co-culture; n = 4 for hAELVi). Statistical significance was assessed using one-way ANOVA followed by Tukey’s *post hoc* test. Significant differences are indicated as follows: *p < 0.05, **p < 0.01, ****p < 0.0001. Detailed statistical results are provided in Supplementary Figure S2. **(B)** Modeling of TEER kinetics using a double sigmoidal nonlinear regression model (Brookes et al., 2021) applied to all three culture conditions. Each point represents the TEER value from an individual insert measured at a given time point. Curves correspond to the double sigmoidal nonlinear regression fitted to the entire dataset for each condition. The model provided excellent fit for hAELVi and hAELVi/MRC-5 cultures, but not for MRC-5 monocultures, consistent with the absence of barrier formation. **(C–E)** Representative immunofluorescence images at day 17 showing intercellular junctional markers in hAELVi epithelial layers: E-cadherin (red, **(C)** Occludin (green, **(D)** and ZO-1 (green, **(E)** DNA is counterstained with Hoechst 33342 (blue). Insets show higher magnification views. Scale bars: 100 μm (main panels), 20 μm (insets).

To assess epithelial barrier establishment over time, transepithelial electrical resistance (TEER) was monitored for up to 28 days in monocultures of hAELVi or MRC-5 cells, as well as in a co-culture model combining both cell types at the air-liquid interface (ALI) (Figure 1A). As expected, MRC-5 fibroblasts failed to develop any measurable resistance throughout the culture period. In contrast, hAELVi monocultures exhibited a steep increase in TEER from day 10 onward, reaching plateau values exceeding 3000 Ω cm^2^ by day 21, indicative of tight junction maturation. The hAELVi/MRC-5 co-culture also showed a progressive rise in TEER, albeit with a slower onset and reaching lower values (∼930 Ω cm^2^ at day 21), supporting barrier formation, albeit with reduced electrical resistance compared to hAELVi monocultures.

To better describe TEER dynamics, all three culture conditions were fitted using a double sigmoidal model adapted from Brooks et al. (Brookes et al., 2021). The model provided a good fit for hAELVi monocultures (adjusted R^2^ = 0.81) and hAELVi/MRC-5 co-cultures (adjusted R^2^ = 0.92), reflecting well-defined biphasic TEER evolution patterns (Figure 1B). In contrast, MRC-5 monocultures exhibited a poor and inconsistent fit, with a negative adjusted R^2^ (−0.02), indicative of the absence of meaningful TEER development. Goodness-of-fit indicators further supported these conclusions: the co-culture condition showed the lowest error values (RMSE = 152.8 Ω cm^2^; Sy.x = 162.3 Ω cm^2^), compared to hAELVi (RMSE = 610.9; Sy.x = 638.7) and MRC-5 (RMSE = 4981; Sy.x = 5292) (Supplementary Table S2). Moreover, residuals passed all normality tests for hAELVi and co-culture models, but not for MRC-5, confirming the robustness of the fit in epithelial conditions only.

Statistical comparisons using one-way ANOVA followed by Tukey’s test indicated that from day 14 onward, TEER was significantly higher in hAELVi cultures compared to both MRC-5 and co-culture conditions. A significant difference between co-culture and MRC-5 conditions was also observed from day 14 to day 28 (detailed p-values are provided in Supplementary Table S1). Of note, no significant difference was observed between the co-culture and MRC-5 monoculture at several time points (e.g., days 14–21). This likely reflects greater variability in co-culture TEER responses and does not contradict the presence of a functional epithelial barrier, as evidenced by junctional protein localization and progressive TEER elevation.

To further characterize the epithelial phenotype at the time of pollutant exposure, immunofluorescence staining of adherent and tight junction proteins was performed at day 17, when TEER values indicated the formation of a functionally relevant barrier. E-cadherin staining showed strong and continuous membrane localization, delineating a cohesive cobblestone-like monolayer typical of polarized alveolar epithelial cells (Figure 1C). In parallel, tight junction proteins ZO-1 and Occludin also displayed well-defined apical localization at intercellular borders (Figures 1D,E), with no major discontinuities or aberrant intracellular accumulation. Consistent with these findings, nuclear staining with Hoechst 33342 further validated cell confluence and monolayer organization. These results confirm the establishment of mature intercellular junctions and support the use of this co-culture model for subsequent exposures under conditions that mimic an intact epithelial barrier.

Altogether, these structural and functional findings validate the use of the hAELVi/MRC-5 co-culture model as a differentiated and polarized alveolar epithelium. Importantly, day 17 was selected as the time point for pollutant exposures based on the attainment of a functionally mature epithelial barrier, as confirmed by TEER plateau values and junctional protein expression.

### 3.2 Physico-chemical characterization of nanoparticular suspensions and dosimetry

**FIGURE 2:**
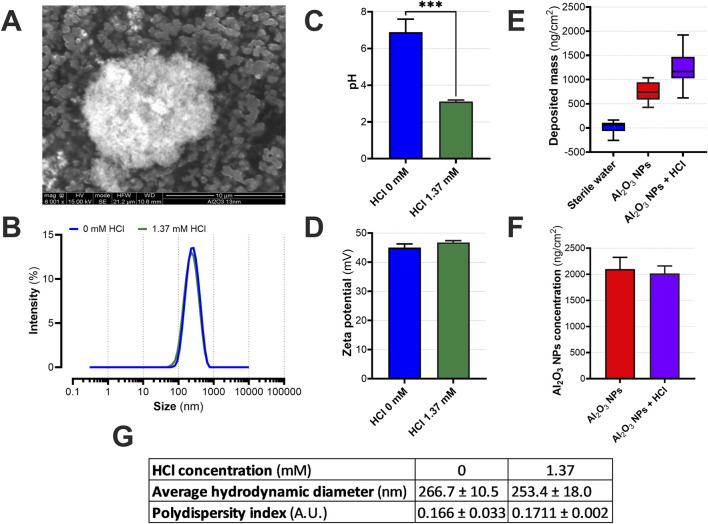
Physico-chemical characterization and dosimetry of Al_2_O_3_ nanoparticle aerosols with or without HCl. **(A)** Scanning electron microscopy (SEM) image of dry agglomerates of Al_2_O_3_ nanoparticles (NPs) prior to aerosolization. **(B)** Size distribution of Al_2_O_3_ NPs suspensions dispersed in sterile water (0 mM HCl, blue) or in the presence of HCl (1.37 mM, green) as measured by dynamic light scattering (DLS); average hydrodynamic diameter and polydispersity index (PDI) are indicated in arbitrary units. **(C)** Measured pH of NPs suspensions before nebulization. **(D)** Zeta potential (mV) of Al_2_O_3_ NPs suspensions in both conditions. **(E)** Deposited mass (ng/cm^2^) on cell inserts at the ALI, quantified in real time using a surface-mounted quartz crystal microbalance (sQCM). **(F)** Total amount of deposited aluminum on co-culture inserts, quantified by Inductively Coupled Plasma Optical Emission Spectrometry (ICP-OES). **(G)** Average hydrodynamic diameters were 266.7 ± 10.5 nm (0 mM HCl) and 253.4 ± 18.0 nm (1.37 mM HCl); PDI values were 0.166 ± 0.033 and 0.171 ± 0.002, respectively. Values represent mean ± SD from three independent experiments. Statistical comparisons were performed using unpaired t-tests, ***p < 0.001.

To characterize the physico-chemical behavior of Al_2_O_3_ nanoparticles (NPs), complementary analyses were conducted on dry material and liquid suspensions prepared in either sterile water or mildly acidic conditions (1.37 mM HCl). SEM imaging of the NPs powder revealed spheroidal primary particles forming dense agglomerates, with overall sizes exceeding several hundred nanometers (Figure 2A). X-ray diffraction (XRD) analysis previously performed by Bourgois et al. (2021) on the same batch of Al_2_O_3_ nanoparticles confirmed the presence of γ and δ crystalline phases, characteristic of transition aluminas generated by combustion processes.

Upon dispersion, both suspensions generated submicron aggregates with comparable hydrodynamic diameters and moderate polydispersity (PDI <0.2) (Figure 2G), and no significant shift in size distribution was observed upon acidification (Figure 2B).

The addition of HCl induced a marked acidification of the medium (from pH 6.89 ± 0.71 to 3.11 ± 0.08, ***p = 0.0008), while the zeta potential remained strongly positive under both conditions (45.00 ± 1.28 mV vs. 46.78 ± 0.63 mV; Figures 2C,D). These high values (>+30 mV) suggest that Al_2_O_3_ NPs maintained their colloidal stability in the presence of acid, consistent with DLVO theory^1^ (Agmo Hernández, 2023).

Deposition efficiency was then assessed under air–liquid interface (ALI) conditions using a Vitrocell® CLOUD-12 exposure system. Gravimetric quantification by surface-mounted quartz crystal microbalance (sQCM) showed minimal mass deposition for control aerosols (sterile water or HCl alone, <500 ng/cm^2^), whereas Al_2_O_3_ NPs nebulized at 1 mg/mL yielded significantly higher deposited masses: 757 ± 210 ng/cm^2^ under neutral conditions and 1224 ± 378 ng/cm^2^ with added HCl (Figure 2E). This increase suggests that acidification may enhance particle delivery to the cell surface.

To verify the actual dose of NPs delivered, total aluminum content was quantified across the insert system by ICP-OES, including the apical hAELVi epithelial layer, the Transwell® membrane, and the underlying MRC-5 fibroblasts. Interestingly, similar quantities of Al_2_O_3_ NPs were detected in both exposure conditions (Figure 2F), indicating that the observed difference in gravimetric deposition does not necessarily translate to a higher bioavailable dose at the cellular level. These findings support the relevance of combining complementary dosimetry approaches to accurately interpret biological outcomes.

Altogether, the demonstrated colloidal stability and controlled, reproducible deposition of Al_2_O_3_ NPs provide a robust physico-chemical foundation for the subsequent biological assessments.

### 3.3 Air-liquid interface exposures to pollutant mixtures do not impair epithelial viability or barrier integrity

To determine whether exposure to pollutant mixtures compromises epithelial viability or barrier function, hAELVi/MRC-5 co-cultures were exposed under air-liquid interface (ALI) conditions using the Vitrocell® CLOUD-12 system. Treatments included sterile water (control), HCl (1.37 mM), Al_2_O_3_ NPs (1 mg/mL), or their combination.

**FIGURE 3:**
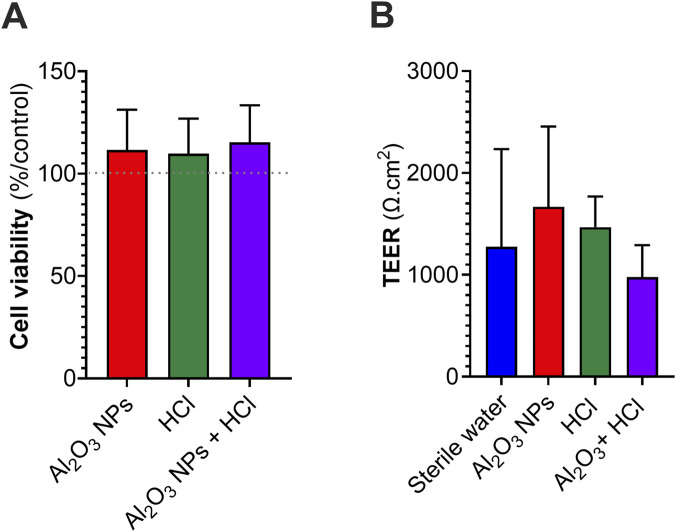
Impact of ALI exposure to Al_2_O_3_ NPs and/or HCl on epithelial barrier integrity and cell viability in hAELVi/MRC-5 co-cultures. hAELVi epithelial cells (apical side) and MRC-5 fibroblasts (basolateral side) were co-cultured on Transwell® inserts and exposed apically under air-liquid interface (ALI) conditions using the Vitrocell® CLOUD-12 system to sterile water, 1.37 mM HCl, Al_2_O_3_ NPs (1 mg/mL), or Al_2_O_3_ NPs +1.37 mM HCl. **(A)** Cell viability of the co-culture was assessed 96 h post-exposure using the PrestoBlue™ metabolic assay. **(B)** Transepithelial electrical resistance (TEER) was measured 96 h after exposure to evaluate barrier integrity. Data are presented as mean ± SD from three independent experiments. Statistical significance was assessed by Kruskal-Wallis with Dunn’s *post hoc* test.

Metabolic activity was assessed 96 h after exposure using the PrestoBlue™ assay. As shown in Figure 3A, no significant difference in viability was observed between the treated groups and the control, suggesting the absence of overt cytotoxicity. Similarly, transepithelial electrical resistance (TEER) measurements (Figure 3B) showed no significant alteration in barrier integrity at 96 h post-exposure, regardless of the treatment.

Statistical analysis using the Kruskal–Wallis test followed by Dunn’s *post hoc* comparisons confirmed the absence of significant effects on both endpoints. Together, these findings indicate that acute ALI exposure to HCl, Al_2_O_3_ NPs, or their combination does not adversely affect the viability or barrier function of the epithelial co-culture model within the studied timeframe.

Apical chloride was stable across experiments (Supplementary Figure S1). Despite background chloride from medium/cells, surrogate doses were comparable between HCl and Al_2_O_3_ + HCl, arguing against random pH artifacts.

### 3.4 Conditioned media of pulmonary cells after ALI exposures to pollutant mixtures increased cell index and cell migration of pulmonary fibroblasts

To assess the paracrine impact of lung cell exposure on fibroblast proliferation, MRC-5 fibroblasts were incubated with conditioned basolateral media (CM) derived from hAELVi monocultures, MRC-5 monocultures, or hAELVi/MRC-5 co-cultures previously exposed at the air-liquid interface (ALI) for 24 h to sterile water, Al_2_O_3_ NPs (1 mg/mL), HCl (1.37 mM), or their combination. Proliferation dynamics were monitored for 53 h using xCELLigence real-time impedance analysis. The baseline-normalized cell index (BNCI) was used to model the response kinetics. All proliferation data were normalized to the reference condition (nebulized sterile water), enabling inter-condition comparisons.

**FIGURE 4:**
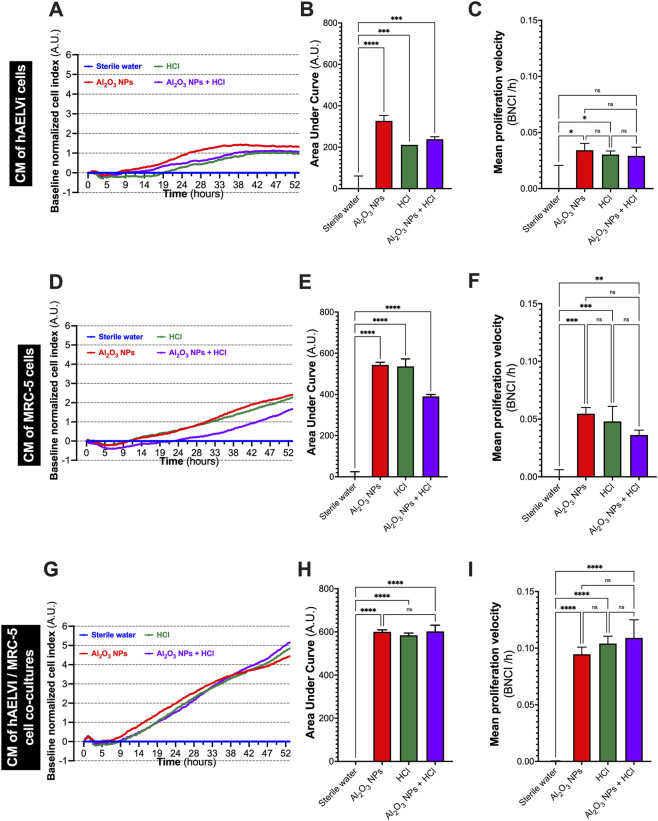
Conditioned media (CM) from exposed lung cells modulate fibroblast proliferation via paracrine mechanisms. MRC-5 fibroblasts were incubated with conditioned media (CM) collected from hAELVi monocultures **(A–C)**, MRC-5 monocultures **(D–F)**, or hAELVi/MRC-5 co-cultures **(G–I)** that had been exposed once daily for four consecutive days at the air–liquid interface (ALI) to sterile water, Al_2_O_3_ NPs (1 mg/mL), HCl (1.37 mM), or their combination. Fibroblast proliferation was continuously monitored over 53 h using the xCELLigence impedance-based system, and the resulting baseline-normalized cell index (BNCI) curves were analyzed to quantify the effects of each condition. **(A,D,G)** Time-course of BNCI showing differential proliferation kinetics depending on the origin of CM and exposure conditions. **(B,E,H)** Area under the curve (AUC) calculated from BNCI curves as an overall measure of proliferative activity. **(C,F,I)** Mean proliferation velocity computed as BNCI per hour. Data are presented as mean ± SD of three independent experiments. Statistical comparisons were performed using one-way ANOVA followed by Tukey’s multiple comparison test. *, **, ***, **** indicate *p* < 0.05, 0.01, 0.001, and 0.0001, respectively; ns: not significant.

CM derived from hAELVi monocultures, MRC-5 monocultures, or hAELVi/MRC-5 co-cultures exposed to Al_2_O_3_ NPs (NPs), HCl, or their combination significantly modulated fibroblast proliferation compared to control CM (Figure 4).

For hAELVi-derived CM (Figures 4A–C), incubation with CM from cells exposed to Al_2_O_3_ NPs or HCl alone resulted in a significant increase in fibroblast proliferation, as shown by higher area under the curve (AUC) and mean proliferation velocity relative to control (*p* < 0.01). In contrast, CM collected after combined Al_2_O_3_ + HCl exposure did not significantly differ from control.

For MRC-5-derived CM (Figures 4D–F), all exposure conditions significantly enhanced proliferation indices compared to control (*p* < 0.0001), with HCl exposure inducing the most pronounced effect on AUC. Mean proliferation velocity was also increased in all exposure groups.

CM from hAELVi/MRC-5 co-cultures **(** Figures 4G–I) induced significantly elevated proliferation in fibroblasts across all tested conditions (*p* < 0.0001), with no marked difference between exposures to Al_2_O_3_, HCl, or their combination.

Overall, these results indicate that soluble mediators released by lung epithelial and mesenchymal cells after exposure to Al_2_O_3_ NPs and/or HCl can promote fibroblast proliferation *in vitro*, with the magnitude of this effect depending on the cell type and the specific exposure. While CM derived from monocultures displayed variable effects, co-culture-derived CM consistently elicited a robust pro-proliferative response regardless of the stimulus.

To further benchmark the effects of Al_2_O_3_ NPs and HCl exposure on fibroblast proliferation and to validate the sensitivity of the experimental system, additional experiments were performed using conditioned media (CM) generated after exposure to canonical profibrotic mediators (TGF-β1 and CTGF) or classical injurious stimuli (bleomycin and LPS). This complementary analysis allowed comparison with well-established models of fibroblast activation and inhibition, thereby reinforcing the interpretability of the observed effects.

Direct exposure to TGF-β1 or CTGF markedly reduced fibroblast proliferation (Supplementary Figures S4A–C). TGF-β1 at 100 pg/mL induced the strongest decline in BNCI, AUC, and proliferation velocity compared to control (*p* < 0.0001). CTGF produced milder but significant inhibition.

CM from hAELVi monocultures exposed to bleomycin or LPS also potently suppressed proliferation (Supplementary Figures S4D–F), with bleomycin showing the most pronounced effects. Similar inhibitory patterns were observed with CM from MRC-5 monocultures (Supplementary Figure S4G–I) and hAELVi/MRC-5 co-cultures (Supplementary Figures S4J–L), both treatments reducing AUC and velocity significantly (*p* < 0.0001).

Overall, these data confirm that classical profibrotic and injurious exposures generate paracrine signals that strongly inhibit fibroblast proliferation, consistent with known effects of TGF-β1, CTGF, bleomycin and LPS on reducing proliferative activity while promoting differentiation or stress responses. This contrasts with the moderate proliferative effects observed after Al_2_O_3_ and HCl exposure.

**FIGURE 5:**
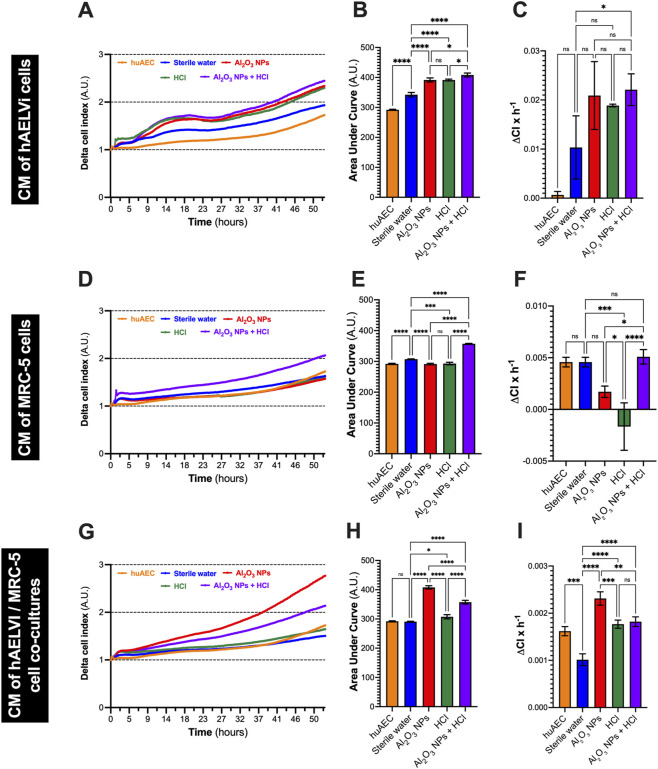
Conditioned media (CM) from exposed lung cells stimulate fibroblast migration via paracrine mechanisms. **(A–C)** Real-time migration kinetics of MRC-5 fibroblasts were monitored over 53 h using an impedance-based system (xCELLigence RTCA; Delta Cell Index, ΔCI) following exposure to CM collected from hAELVi cells **(A)**, with corresponding quantifications of area under the curve (AUC) values extracted from the ΔCI kinetic profiles, representing cumulative cell migration over the 53 h period, **(B)** and migration velocity (ΔCI × h^-1^) calculated from second-order polynomial regression models fitted to each condition’s time-course data **(C)**. This parameter reflects the average migration rate over the full monitoring window. **(D–F)** Same experimental setup using CM from MRC-5 fibroblasts as donor cells: migration velocity kinetics **(D)**, AUC **(E)**, and calculated migration velocity **(F)**. **(G–I)** Same analysis using CM from hAELVi/MRC-5 co-cultures exposed under identical conditions: migration velocity kinetics **(G)**, AUC **(H)**, and migration velocity **(I)**. Donor cells had been exposed under air–liquid interface (ALI) conditions to sterile water (vehicle control), Al_2_O_3_ NPs (1 mg/mL), HCl (1.37 mM), or their combination. Unconditioned medium (huAEC) served as baseline control. Data are expressed as mean ± SD. n = 4 independent CM preparations for the huAEC condition, and n = 3 for all other exposure conditions. Statistical significance was assessed using one-way ANOVA followed by Tukey’s *post hoc* test. *p* < 0.05 (*), <0.01 (**), <0.001 (***), <0.0001 (****); ns: not significant.

To determine whether ALI-exposed lung cells release paracrine signals capable of modulating fibroblast migration, we collected conditioned media (CM) from hAELVi, MRC-5, or hAELVi/MRC-5 co-cultures 24 h post-exposure and applied them to the lower chambers of CIM-plates seeded with MRC-5 fibroblasts (Figures 5A,D,G). Migration was monitored in real time using impedance-based measurements (xCELLigence™). CM from hAELVi cells previously exposed to HCl, Al_2_O_3_ NPs, or their combination induced a moderate but reproducible increase in fibroblast migration compared to unconditioned media (huAEC), with the strongest effect observed for the Al_2_O_3_ + HCl mixture (Figure 5A). This translated into significantly higher AUC values (Figure 5B), although no significant increase in migration velocity (ΔCI/h) was detected except for the combination condition (Figure 5C).

Interestingly, CM derived from exposed MRC-5 fibroblasts alone induced only a weak enhancement in migration velocity kinetics (Figure 5D), with marginal differences in AUC (Figure 5E) and no significant effect on migration velocity across conditions (Figure 5F). In contrast, when CM originated from exposed co-cultures, fibroblast migration was markedly increased (Figure 5G), especially after exposure to Al_2_O_3_ NPs or Al_2_O_3_ + HCl, with significant rises in both AUC (Figure 5H) and migration velocity (Figure 5I). These findings suggest that fibroblast activation is primarily driven by soluble signals released in the context of epithelial-mesenchymal interaction, rather than by epithelial or mesenchymal cells in isolation.

To validate the responsiveness of the migration assay, we first tested the effect of recombinant TGF-β1 and CTGF applied at 10 or 100 pg/mL directly in the donor compartment of CIM-plates. As shown in Supplementary Figures S5A–C, both cytokines induced a dose-dependent increase in fibroblast migration, reflected in significantly higher AUC values and increased migration velocities. These results confirm the functional sensitivity of the system to canonical profibrotic cues.

We then investigated whether classical inflammatory or fibrotic stimuli such as LPS or bleomycin could replicate the effects of ALI exposure. CM were collected from hAELVi, MRC-5, or hAELVi/MRC-5 co-cultures exposed under submerged conditions and applied to the CIM-plates. CM from LPS-exposed hAELVi cells induced a slight increase in migration (Supplementary Figure S5C–E), while bleomycin had no significant effect. Similarly, CM from MRC-5 monocultures did not significantly modify migration profiles (Supplementary Figure S5F–H). In contrast, CM from co-cultures exposed to either LPS or bleomycin moderately increased AUC values compared to huAEC (Supplementary Figure S5J), although only LPS significantly altered the migration kinetics (Supplementary Figure S5K), suggesting a possible shift in the temporal dynamics of cell displacement.

Together, these results demonstrate that ALI exposure to Al_2_O_3_ and HCl triggers the release of soluble factors that can promote fibroblast migration in a donor-dependent manner. The most robust effects were observed when conditioned media originated from co-cultures, highlighting the importance of epithelial–mesenchymal interactions in shaping the paracrine response. Furthermore, the fact that both canonical (TGF-β1, CTGF) and environmental stimuli (LPS, bleomycin) elicited similar patterns - albeit with variable intensity - underscores the relevance of this model to detect diverse classes of profibrotic signals. These findings suggest that multiple exposure scenarios can lead to the emergence of soluble paracrine signals capable of modulating fibroblast behavior, even in the absence of direct cell-cell contact. The molecular nature of this signal remains to be investigated.

To determine whether fibroblast proliferation and migration were co-regulated in response to conditioned media (CM) derived from pollutant-exposed lung cells, Pearson correlation analyses were performed using the mean proliferation velocity (BNCI/h) and mean migration velocity (ΔCI/h) measured by real-time impedance assays.

**FIGURE 6:**
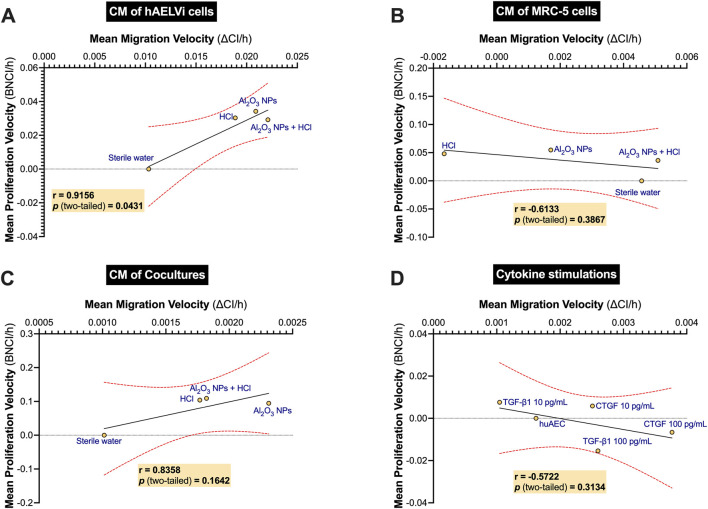
Correlation between fibroblast migration and proliferation in response to conditioned media from exposed lung cells and recombinant cytokines. Pearson correlation analyses between mean migration velocity (ΔCI/h; X-axis) and mean proliferation velocity (BNCI/h; Y-axis) of MRC-5 fibroblasts incubated with conditioned media (CM) collected from **(A)** hAELVi monocultures, **(B)** MRC-5 monocultures, and **(C)** hAELVi/MRC-5 co-cultures exposed for 24 h at the air–liquid interface (ALI) to sterile water, Al_2_O_3_ NPs (1 mg/mL), HCl (1.37 mM), or their combination. Panel **(D)** shows correlations for fibroblasts directly treated with recombinant TGF-β1 or CTGF at 10 or 100 pg/mL. Each point represents the mean value for a given condition from three to four independent experiments. Solid black lines indicate linear regression fits, with red dashed lines representing the 95% confidence intervals. Pearson’s correlation coefficients (r) and corresponding two-tailed *p*-values are reported in each panel.

When CM originated from hAELVi monocultures (Figure 6A), a strong positive correlation was observed between proliferation and migration velocities (r = 0.9156, *p* = 0.0431), suggesting that epithelial-derived soluble factors can coordinately stimulate both processes.

In contrast, CM from MRC-5 monocultures (Figure 6B) showed a moderate negative correlation (r = - 0.6133) that did not reach statistical significance (*p* = 0.3867), indicating a decoupling between proliferation and migration in this context.

For CM derived from hAELVi/MRC-5 co-cultures (Figure 6C), a strong positive correlation was again detected (r = 0.8358), though this association was not statistically significant (*p* = 0.1642).

Finally, when fibroblasts were exposed to recombinant TGF-β1 or CTGF (Figure 6D), no significant correlation was observed (r = −0.5722, *p* = 0.3134).

Altogether, these analyses demonstrate that the coordinated stimulation of fibroblast proliferation and migration is not a universal feature of profibrotic or inflammatory signals but appears specific to soluble mediators released by pollutant-exposed epithelial cells, highlighting the unique paracrine signature of epithelial stress responses.

### 3.5 Pollutant exposures do not elicit major inflammatory or fibrotic secretory responses: insights from cytokine array and ELISA

To assess whether pollutant exposure triggers the release of inflammatory or fibrotic mediators, we performed a semi-quantitative analysis of 105 soluble proteins in basolateral media collected 96 h after ALI exposure of hAELVi/MRC-5 co-cultures. Treatments included Al_2_O_3_ NPs (1 mg/mL), HCl (1.37 mM), their combination, and sterile water as control. Additional co-cultures exposed to bleomycin (0.15 μg/mL) and lipopolysaccharide (LPS, 1 μg/mL) served as pro-fibrotic and pro-inflammatory positive controls, respectively.

**FIGURE 7:**
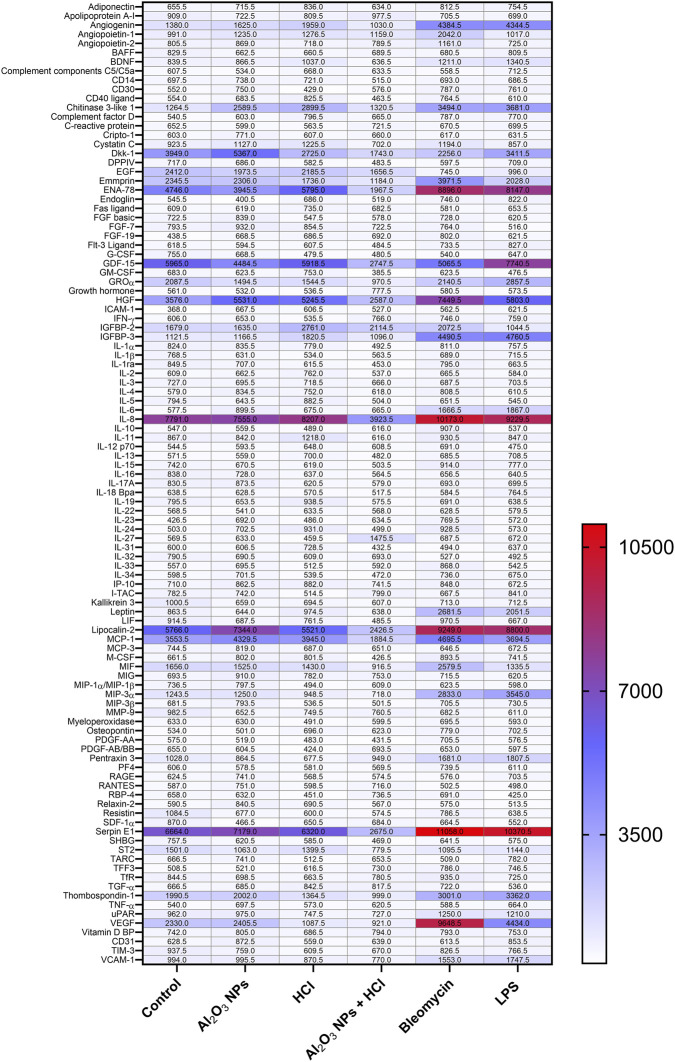
Cytokine secretion profile in co-culture baso-lateral medium following ALI exposure to Al_2_O_3_ NPs and/or HCl. hAELVi/MRC-5 co-cultures were exposed apically under air-liquid interface (ALI) conditions to sterile water (control), 1.37 mM HCl, Al_2_O_3_ NPs (1 mg/mL), or Al_2_O_3_ NPs +1.37 mM HCl using the Vitrocell® CLOUD-12 system. Additional co-cultures were apically exposed to lipopolysaccharide (LPS, 1 μg/mL) or bleomycin (0.15 μg/mL) as pro-inflammatory and pro-fibrotic positive controls, respectively. After 96 h, basolateral medium (supernatant recovered from the lower compartment)were collected, and for each condition, a pool of three Transwell® inserts was analyzed. Secreted cytokines were profiled using the Proteome Profiler™ XL Human Cytokine Array Kit (R&D Systems/Bio-Techne), enabling the simultaneous semi-quantitative detection of 105 cytokines, chemokines, and growth factors. The heatmap shows relative signal intensities of selected proteins normalized to the control condition, highlighting differential expression patterns across treatments. Because this array is semi-quantitative, results are descriptive and not subjected to statistical testing. Data shown are representative of three independent experiments Supplementary Material.

The resulting heatmap (Figure 7) shows normalized signal intensities for selected cytokines, chemokines, and growth factors. Exposure to Al_2_O_3_ NPs, HCl, or their mixture induced no substantial increase in the secretion of classical pro-inflammatory cytokines (e.g., IL-6, IL-8, TNF-α), chemokines (e.g., MCP-1, ENA-78), or fibrotic mediators (e.g., TGF-β1, PDGF, CTGF), in contrast to the marked induction observed with LPS and, to a lesser extent, with bleomycin. Interestingly, in some cases (e.g., IL-8, Lipocalin-2, MCP-1), the Al_2_O_3_ NPs + HCl condition resulted in lower cytokine signal intensity compared to single exposures or even control, suggesting a potential dampening rather than additive effect. These results suggest that pollutant-exposed co-cultures do not display a strong secretory response under these conditions. As the cytokine array is semi-quantitative, these observations mainly indicate trends in mediator modulation.

To support the protein array findings, we further quantified selected secreted factors (IL-8, TGF-β1, CTGF, Collagen I) by ELISA (see Supplementary Figure S6). The data confirmed the absence of significant upregulation following exposure to pollutant mixtures, thereby reinforcing the notion of limited canonical paracrine signaling in this model. Although not statistically significant, a trend toward increased IL-8 secretion can be noted for both LPS and bleomycin exposures.

## 4 Discussion

In this study, we established a physiologically relevant air-liquid interface (ALI) co-culture model combining human alveolar epithelial (hAELVi) and lung fibroblast (MRC-5) cells to investigate the early cellular responses to repeated, sub-lethal exposures to aluminum oxide nanoparticles (Al_2_O_3_ NPs) and hydrogen chloride (HCl). This model allowed us to mimic realistic co-exposure scenarios encountered in industrial or military contexts while monitoring both epithelial integrity and paracrine signaling.

Our main findings are that: (i) exposures to Al_2_O_3_ NPs, HCl, or their mixture did not cause overt cytotoxicity or barrier leakage; (ii) fibroblast proliferation and migration were nonetheless modulated by conditioned media from exposed epithelial or co-cultures; and (iii) these paracrine effects occurred in the absence of canonical profibrotic mediators such as TGF-β1 or CTGF, suggesting the involvement of non-canonical epithelial-mesenchymal signaling pathways. Together, these results identify early, cytokine-independent fibroblast activation as a potential initiating event in pollutant-induced lung remodeling.

Interestingly, the paracrine effects observed on fibroblast proliferation and migration were consistently stronger when the conditioned media originated from co-cultures compared to monocultures. This suggests that the epithelial–mesenchymal crosstalk established in co-culture may generate unique soluble signals that are absent or less pronounced in monocultures. Such enhanced effects could reflect synergistic interactions between epithelial-derived stress signals and fibroblast responses, potentially mediated by bidirectional signaling loops, enhanced metabolic cooperation, or the release of composite extracellular vesicles containing factors from both cell types. This strengthens the argument that advanced multicellular models are required to capture emergent properties of intercellular communication that cannot be reproduced in monocultures.

Methodologically, the air–liquid interface (ALI) co-culture model combining alveolar epithelial and lung fibroblast cell lines offers a physiologically relevant platform to assess early responses to inhaled substances. Compared to conventional submerged systems, ALI cultures better replicate key features of the distal lung epithelium, including barrier formation, junctional protein expression, and stress responses upon aerosol exposure (Sengupta et al., 2022; Clippinger et al., 2016). This model enables direct delivery of airborne pollutants to the apical surface of epithelial cells, leading to more realistic biological responses such as oxidative stress, cytokine production, and loss of barrier integrity (Upadhyay and Palmberg, 2018; Lenz et al., 2013). Such characteristics make the model particularly relevant for regulatory toxicology, where capturing early “silent” responses may improve hazard identification.

Although TEER values in the hAELVi/MRC-5 co-culture were consistently higher than those in MRC-5 monocultures from day 17 onward, the difference did not reach statistical significance at certain time points (e.g., D17 to D21), likely due to inter-experimental variability and the limited sample size (n = 3). Nonetheless, the magnitude of the difference and the consistent trend across time points support the interpretation of effective barrier formation in the co-culture model. This stable epithelial barrier is essential for interpreting paracrine effects as genuine signaling events rather than artifacts of epithelial leakage.

However, ALI models have notable limitations. They lack the microvascular and immune components of the lung environment, which are essential for studying epithelial-endothelial crosstalk and immune cell-mediated fibrosis (Huh et al., 2010; Rock et al., 2011). While the ALI co-culture recapitulates epithelial–mesenchymal crosstalk, it lacks immune and endothelial components that are essential for modeling *in vivo* inflammation and vascular remodeling. Integrating macrophages or microvascular endothelial cells into multicellular ALI or lung-on-chip systems represents a logical next step to enhance the physiological and predictive relevance of this model. This is an important limitation, since these immune cells are central to pollutant clearance and the initiation of inflammatory cascades. Future studies should therefore integrate macrophages and other immune populations into multicellular ALI models, in order to better recapitulate the complex interplay driving lung responses to mixed exposures. Without macrophages or neutrophils, key aspects of the innate immune response to inhaled substances cannot be captured. A limitation of our exposure design is the use of 1.37 mM HCl, which generates a markedly acidic environment (pH ∼3). While this may exceed typical environmental exposure levels, it was intended as a sub-lethal but biologically relevant insult *in vitro*. For translational reference, the cumulative deposited mass of Al_2_O_3_ NPs after four daily nebulizations was estimated at approximately 1.1 μg/cm^2^, based on quartz crystal microbalance (sQCM) and ICP-OES quantification. This dose lies within the sub-lethal range commonly used in ALI studies and corresponds to surface burdens measured in short-term occupational inhalation scenarios (Clippinger et al., 2016). For HCl, the applied 1.37 mM solution approximates 50 ppm in gaseous form, reflecting acute accidental or confined-space exposures (Colunga Biancatelli et al., 2021). These levels were therefore selected to probe mechanistic rather than hazard-based responses, ensuring early paracrine effects could be detected without overt cytotoxicity. These findings, together with our chloride quantification, indicate controlled and comparable acid delivery across HCl and Al_2_O_3_ NPs + HCl mixtures. Furthermore, in our previous work (Bourgois et al., 2019) we showed that HCl at 1.1 mM did not induce cytotoxicity in submerged A549 cultures after 24 h (PrestoBlue® viability assay) or 48 h (xCELLigence real-time follow-up), supporting that the present ALI responses are not explained by non-specific acid toxicity. Moreover, while extrapolation to humans remains limited, prior work on combustion gases showed that *in-vitro* IC_50_ values in A549 or skin fibroblasts correlate with human Lowest Concentration/Lowest Dose causing lethality (LCLo/LDLo) when an adjustment factor is applied (Lestari et al., 2005), supporting the translational relevance of sub-lethal *in-vitro* readouts. Future studies should include dose–response assessments with lower HCl concentrations to better align with variable human exposure scenarios.

A key limitation of this study is the use of a single exposure level. This choice was motivated by the mechanistic scope of the work, which aimed to characterize early epithelial-fibroblast communication under a realistic, non-cytotoxic condition. Follow-up studies will introduce multi-dose designs to assess quantitative activation thresholds.

In addition, the absence of a perfusable vascular network limits the ability to study systemic dissemination or clearance of toxicants. Thus, while our findings highlight early epithelial-fibroblast communication, they should be considered as a minimal system that may underestimate the contribution of immune or vascular signals.As illustrated in Figure 4B, conditioned media from exposed MRC-5 monocultures significantly increased proliferation, especially after exposure to HCl, reinforcing the idea that fibroblast behavior is influenced by donor cell type and exposure context. These findings reinforce the concept that alveolar epithelial cells actively regulate the behavior of surrounding stromal cells, contributing to early tissue remodeling. This is consistent with the growing recognition that epithelial-mesenchymal crosstalk plays a critical role in fibrotic initiation, even in the absence of classical profibrotic mediators such as TGF-β1 or CTGF (Kendall and Feghali-Bostwick, 2014; Pardo and Selman, 2016). Recent studies have shown that epithelial injury alone can prime resident fibroblasts toward a profibrotic phenotype through mechano-chemical cues and altered extracellular matrix (ECM) dynamics, independently of inflammation (Rock et al., 2011; Hinz et al., 2007; King et al., 2011).

Because the cytokine array used is semi-quantitative, these results should be interpreted with caution. The absence of strong cytokine induction suggests - but does not conclusively demonstrate - that fibroblast activation occurred through non-canonical, cytokine-independent mechanisms. Follow-up quantitative assays and vesicle fractionation will be necessary to confirm this interpretation. Crucially, the absence of significant increases in inflammatory cytokines such as IL-8 and IL-6 supports the hypothesis that non-canonical pathways are involved in the early activation of fibroblasts, a response commonly associated with acute epithelial injury (Chen et al., 2020). Interestingly, no synergistic effects were observed when combining Al_2_O_3_ NPs and HCl, as the responses in terms of fibroblast migration and proliferation were comparable to those induced by single exposures. This absence of synergy may reflect the activation of independent and possibly non-overlapping pathways by NPs and acidic gases. In such cases, mixture effects can be neutral rather than additive or synergistic, as commonly reported in inhalation toxicology where interactions between pollutants may vary from synergistic to antagonistic depending on the context. These observations are consistent with our finding that co-exposure did not amplify fibroblast responses compared to single exposures. Our results therefore highlight the complexity of mixture toxicology and suggest that co-exposures do not necessarily exacerbate early paracrine signaling beyond the effects of individual components.

While our study clearly demonstrates that sub-lethal exposures to Al_2_O_3_ and HCl can elicit early paracrine effects on fibroblasts, it is important to acknowledge that the 96-h observation window only captures short-term epithelial–mesenchymal communication. Hallmark fibrotic features such as sustained TGF-β1 release, collagen deposition, or α-SMA induction (Kendall and Feghali-Bostwick, 2014; Hinz et al., 2007) typically emerge after extended periods (≥7–14 days) *in vitro* or following chronic exposures *in vivo* (Kadota et al., 2020; Yao et al., 2024). Therefore, our findings should be interpreted as evidence of early remodeling cues rather than definitive proof of fibrogenesis. Future studies including longer follow-up or repeated exposures will be essential to determine whether the observed fibroblast activation evolves into persistent fibrotic remodeling.

Recent evidence indicates that epithelial–mesenchymal communication can also rely on non-canonical mediators such as extracellular vesicles (EVs), DAMPs, or metabolic cues (Kadota et al., 2020; Lacedonia et al., 2021). While our data cannot identify specific mediators, the combination of absent cytokine induction and measurable fibroblast activation is compatible with such alternative pathways.In line with our observation that canonical mediators such as TGF-β1, CTGF, and IL-8 were not significantly altered, our data support the hypothesis that fibroblast activation may rely on non-canonical paracrine mechanisms. These findings fit within the Adverse Outcome Pathway (AOP) framework for pulmonary fibrosis, in which fibroblast activation and proliferation represent an early key event. The lack of canonical cytokine induction suggests that alternative mediators, such as extracellular vesicles (EVs), damage-associated molecular patterns (DAMPs) like HMGB1, or metabolic stress signals (e.g., lactate, ATP, succinate), may transmit epithelial stress signaling independently of classical profibrotic cytokines. Future work involving inhibition of vesicle release or targeted metabolomic profiling will help identify these non-canonical pathways. Several alternative pathways have been proposed in the literature, including the release of extracellular vesicles (EVs) enriched in microRNAs, proteins, or lipids, as well as metabolic mediators such as lactate, ATP, or other stress-associated metabolites. Future studies will be necessary to experimentally test these hypotheses, for instance by using pharmacological inhibitors of EV secretion (e.g., GW4869) (Essandoh et al., 2015; Kim et al., 2022) or by fractionating conditioned media according to molecular weight to distinguish vesicular from soluble signals (Akers et al., 2013; Lobb et al., 2015). In addition, targeted metabolomic profiling (e.g., LC-MS/MS) or small RNA sequencing could help identify specific metabolites or regulatory microRNAs involved in fibroblast migration and proliferation. Such approaches will provide mechanistic insight into cytokine-independent signaling and may uncover novel biomarkers of early lung remodeling.

Beyond vesicles and protein mediators, epithelial-derived metabolites may also contribute to paracrine signaling. Bioactive metabolites like lactate and extracellular ATP, often released under metabolic stress, have been implicated in promoting fibroblast activation and myofibroblast differentiation (Kottmann et al., 2012; Xie et al., 2016). These so-called “danger co-mediators” might act in concert with DAMPs “Damage-Associated Molecular Patterns” to initiate tissue remodeling independently of classical inflammatory pathways. Applying metabolomic profiling approaches such as LC-MS/MS or NMR spectroscopy (Wishart, 2016; Emwas et al., 2013), coupled with stable isotope tracing (Jang et al., 2018), could help elucidate the origin and role of these metabolic signals. Notably, several circulating metabolites such as lactate, succinate, and kynurenine have been proposed as potential biomarkers of fibrotic lung diseases, including idiopathic pulmonary fibrosis and systemic sclerosis-associated interstitial lung disease (Kottmann et al., 2012; Pan et al., 2024; Summer et al., 2024). Investigating whether similar metabolites are secreted under mixed Al_2_O_3_ + HCl exposures could provide important mechanistic insight.

These findings reinforce the concept that alveolar epithelial cells actively regulate the behavior of surrounding stromal cells and can contribute to early remodeling processes. This is consistent with the growing recognition that epithelial-mesenchymal crosstalk plays a central role in the initiation of fibrotic responses, even before the appearance of classical profibrotic mediators such as TGF-β1 or CTGF (Kendall and Feghali-Bostwick, 2014; Pardo and Selman, 2016). In particular, evidence indicates that fibroblast priming can occur via alternative, non-canonical pathways. Recent work has shown that epithelial injury alone can prime resident fibroblasts toward a profibrotic phenotype through mechano-chemical cues and altered ECM dynamics, even in the absence of inflammation (Rock et al., 2011; Hinz et al., 2007; King et al., 2011). The absence of significant increases in inflammatory cytokines like IL-8 or IL-6 further suggests that the signals involved may differ from those typically observed during acute injury (Chen et al., 2020). Recent literature points to extracellular vesicles and microRNAs as potential mediators of intercellular communication under such conditions. These vesicles, secreted by stressed epithelial cells, can carry bioactive molecules such as microRNAs or metabolic regulators capable of modulating fibroblast proliferation, migration, and differentiation (Kadota et al., 2020; Lacedonia et al., 2021). Taken together, our results support the hypothesis that vesicle-associated and cytokine-independent signals are key contributors to the observed fibroblast activation.

Furthermore, exposure to nanoparticles has been shown to induce sub-lethal stress, including redox imbalance, and mitochondrial perturbation, which may lead to the release of endogenous danger signals or DAMPs (Sayan and Mossman, 2016; Baron et al., 2015). DNA damage in epithelial cells, even in the absence of overt cytotoxicity, has been shown to alter their secretory profile, thereby influencing the surrounding microenvironment. Specifically, a senescence-associated secretory phenotype (SASP), or a SASP-like profile, may emerge, characterized by the release of pro-inflammatory cytokines (e.g., IL-6, IL-8), growth factors, DAMPs, and extracellular vesicles (Coppé et al., 2008; Ohtani, 2022). These factors are known to modulate fibroblast behavior, promoting their proliferation, migration, and pro-fibrotic potential. Among these DAMPs, HMGB1 and extracellular ATP have been shown to activate fibroblasts via inflammasome-independent pathways (Rubartelli and Lotze, 2007; Thannickal et al., 2014), suggesting their role as early fibrogenic cues. Collectively, these alternative signaling routes are now increasingly recognized as central to the priming of mesenchymal cells for fibrotic remodeling, even in the absence of overt inflammation (Sharma et al., 2018).

In our model, the combination of Al_2_O_3_ NPs and HCl did not consistently enhance paracrine activation compared to each pollutant alone. This absence of clear synergistic effects suggests that co-exposure may lead to complex, non-additive interactions in epithelial secretory responses. Indeed, acidic environments are known to increase epithelial barrier permeability and oxidative stress responses, thereby enhancing sensitivity to inhaled particles (Longhin et al., 2013; Oberdörster et al., 2005). Previous studies have shown that acidic environments can increase epithelial vulnerability to particulate matter (Colunga Biancatelli et al., 2021; Ohwada et al., 2003). In our model, however, co-exposure did not further amplify fibroblast responses beyond those observed with single exposures. This highlights that mixed exposures do not necessarily produce additive effects but may instead generate context-dependent outcomes, which need to be carefully assessed in experimental and regulatory toxicology. Although combustion or accidental releases may involve higher acute concentrations, the chosen cumulative dose (∼1.1 μg/cm^2^, measured by ICP-OES after four daily nebulizations) is consistent with previous *in vitro* and inhalation studies in rodents (Bourgois et al., 2019; Bourgois et al., 2021) and falls within a sub-lethal range appropriate to investigate early paracrine responses.

## 5 Conclusion

Importantly, our results suggest that fibroblast activation can occur independently of the classical TGF-β1/CTGF axis, commonly regarded as a hallmark of fibrogenesis (Byrne and Baugh, 2008; Bagnato and Harari, 2015). This indicates that early stages of fibrotic remodeling may be driven by alternative signaling pathways and contact-independent cues.

We observed that ALI exposure to pollutant mixtures elicited variable effects on fibroblast proliferation and a consistent enhancement of migration, depending on the exposure and the cellular source of conditioned media, even in the absence of canonical cytokine induction. This provides direct evidence that atypical epithelial-mesenchymal communication can prime fibroblasts through non-canonical mechanisms.

Together, these findings point to the emergence of non-classical paracrine signals, shaped by epithelial–mesenchymal interactions at the alveolar barrier, that may initiate fibrotic remodeling before overt inflammation becomes detectable.

From a toxicological and regulatory perspective, these results highlight that sub-lethal exposures can elicit atypical paracrine signals not captured by conventional endpoints such as cytotoxicity or IL-6/IL-8 release. Incorporating such early markers, including fibroblast migration, into advanced ALI co-culture systems could improve the predictive value of *in vitro* assays for risk assessment of complex aerosols (Clippinger et al., 2016; Halappanavar et al., 2023).

Further investigations should aim to identify the nature of the soluble mediators involved - potentially unconventional cytokines, metabolites, or extracellular vesicles - through fractionation, proteomic profiling, or pharmacological inhibition.

It would also be informative to extend exposure durations, incorporate immune cells, and assess the evolution of paracrine signaling under repeated or chronic exposures. Integrating such approaches with advanced 3D models and biomarker discovery pipelines could help bridge early *in vitro* findings with clinically relevant endpoints, thereby improving the assessment of complex airborne exposures.

## Data Availability

The raw data supporting the conclusions of this article will be made available by the authors, without undue reservation.

## Footnotes

1 Derjaguin–Landau–Verwey–Overbeek theory.
