# Supplementary material for: Beyond cytotoxicity: pollutant mixtures elicit unconventional epithelial-fibroblast signaling in a human lung air-liquid interface co-culture model
Source: Front Toxicol. 2025 Dec 18;7:1722968. doi: 10.3389/ftox.2025.1722968 (PMC12756146; doi:10.3389/ftox.2025.1722968)
Supplement: Supplementary file 1 [file DataSheet1.docx]

Supplementary Material


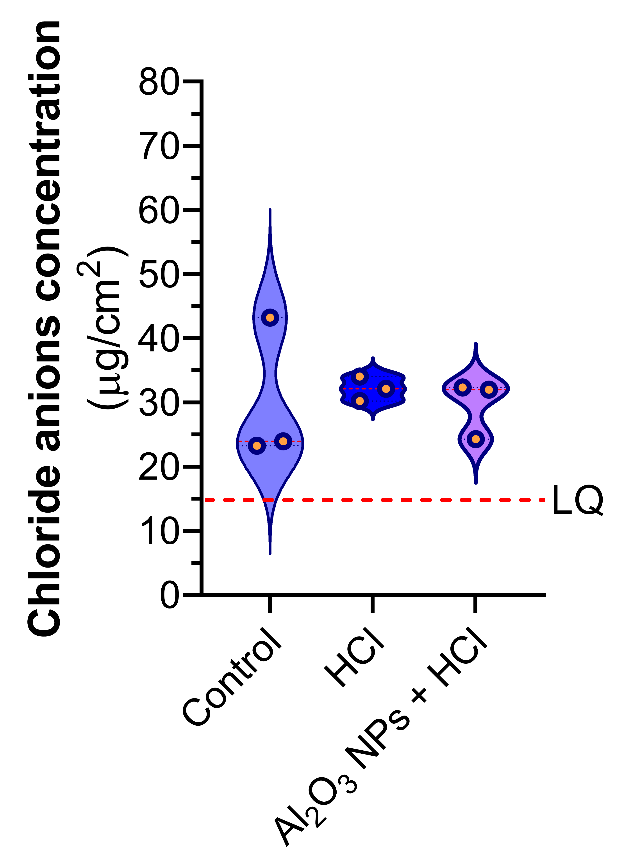


| **Condition** | **Cl⁻ (µg/insert)** (indiv.) | **Mean ± SD** | **µg/cm²**  **(mean ± SD)** | **H⁺ eq. (nmol/cm²)**  **(mean ± SD)** |
| --- | --- | --- | --- | --- |
| Control | 26.09, 26.84, 48.42 | **33.79 ± 12.68** | **30.17 ± 11.32** | **850.9 ± 319.4** |
| HCl | 38.13, 33.87, 36.00 | **36.00 ± 2.13** | **32.14 ± 1.90** | **906.7 ± 53.7** |
| Al₂O₃ NPs + HCl | 36.26, 27.22, 35.88 | **33.12 ± 5.11** | **29.57 ± 4.56** | **834.1 ± 128.7** |

**Figure S1. Apical chloride quantification after ALI exposures measured according to NF EN ISO 10304 (2009).** Violin plots with individual data points (n = 3 inserts/condition) show chloride mass per insert (µg/insert) measured in apical rinsates after exposure to sterile water (Control), HCl (1.37 mM), or Al₂O₃ NPs (1 mg/mL) + HCl (1.37 mM). The red dashed horizontal line marks the quantification limit (LQ = 0.15 µg/insert); values below LQ were treated as non-quantifiable. For dose reporting in the text, data were also converted to µg/cm² (insert area 1.12 cm²) and acidity-equivalent nmol H⁺/cm² assuming 1:1 stoichiometry (HCl → H⁺ + Cl⁻; 35.45 µg Cl⁻ = 1 µmol). Individual values and summary statistics are provided in the accompanying supplementary table.

| Time (days) | Comparison | Mean difference (Ω.cm^2^) | 95% CI | Adjusted *p-value* | Significance |
| --- | --- | --- | --- | --- | --- |
| 14 | MRC-5 vs hAELVi | –1140 | –1948 to –332.6 | 0.0032 | ** |
|  | MRC-5 vs hAELVi/MRC-5 | –313.7 | –1177 to 549.5 | 0.6625 | ns |
|  | hAELVi vs hAELVi/MRC-5 | 826.4 | 18.93 to 1634 | 0.0437 | * |
| 17 | MRC-5 vs hAELVi | –2131 | –2938 to –1323 | < 0.0001 | **** |
|  | MRC-5 vs hAELVi/MRC-5 | –548.9 | –1412 to 314.3 | 0.2881 | ns |
|  | hAELVi vs hAELVi/MRC-5 | 1582 | 714.5 to 2389 | < 0.0001 | **** |
| 19 | MRC-5 vs hAELVi | –2680 | –3488 to –1873 | < 0.0001 | **** |
|  | MRC-5 vs hAELVi/MRC-5 | –785.6 | –1652 to 104.7 | 0.0966 | ns |
|  | hAELVi vs hAELVi/MRC-5 | 1922 | 1114 to 2729 | < 0.0001 | **** |
| 21 | MRC-5 vs hAELVi | –3049 | –3857 to –2242 | < 0.0001 | **** |
|  | MRC-5 vs hAELVi/MRC-5 | –816.5 | –1680 to 46.70 | 0.0677 | ns |
|  | hAELVi vs hAELVi/MRC-5 | 2233 | 1425 to 3040 | < 0.0001 | **** |
| 24 | MRC-5 vs hAELVi | –2587 | –3395 to –1780 | < 0.0001 | **** |
|  | MRC-5 vs hAELVi/MRC-5 | –1059 | –1932 to –205.6 | 0.0112 | ** |
|  | hAELVi vs hAELVi/MRC-5 | 1519 | 711.0 to 2326 | < 0.0001 | **** |
| 26 | MRC-5 vs hAELVi | –3466 | –4273 to –2658 | < 0.0001 | **** |
|  | MRC-5 vs hAELVi/MRC-5 | –1340 | –2203 to –476.9 | 0.0011 | ** |
|  | hAELVi vs hAELVi/MRC-5 | 2126 | 1318 to 2933 | < 0.0001 | **** |
| 28 | MRC-5 vs hAELVi | –3411 | –4218 to –2603 | < 0.0001 | **** |
|  | MRC-5 vs hAELVi/MRC-5 | –1707 | –2571 to –844.2 | < 0.0001 | **** |
|  | hAELVi vs hAELVi/MRC-5 | 1703 | 895.7 to 2511 | < 0.0001 | **** |

**Figure S2. Statistical comparisons of TEER values between culture conditions at each time point.** One-way ANOVA followed by Tukey’s multiple comparisons test was performed to assess differences in TEER between hAELVi monocultures, MRC-5 monocultures, and hAELVi/MRC-5 co-cultures at each time point from day 14 to day 28. Reported *p-values* were adjusted for multiple comparisons using Tukey’s honestly significant difference (HSD) procedure. The table shows mean differences, 95% confidence intervals (CI), adjusted *p-values*, and corresponding significance levels. Significant differences are indicated as follows: *p* < 0.05 (*), *p* < 0.01 (**), *p* < 0.001 (***), *p* < 0.0001 (****). Comparisons that did not reach statistical significance are labeled as “ns”. Non-significant results between MRC-5 and co-culture groups at certain time points (e.g., D17–D21) may reflect high variability and limited statistical power, despite consistent TEER trends.

| Cell model : | MRC-5 | hAELVi | hAELVi/MRC-5 |
| --- | --- | --- | --- |
| Best-fit values |  |  |  |
| A | 9.78 | 3249 | 3045 |
| k_1_ | 0.1912 | 0.4271 | Unstable |
| t_1_ | 3.464 | 15.47 | Unstable |
| k_2_ | 0.6472 | Unstable | -0.1641 |
| t_2_ | 31.57 | Unstable | 26.94 |
| Adjusted R^2^ | -0.02353 | 0.8135 | 0.9172 |
| RMSE (Ω.cm^2^) | 4.981 | 610.9 | 152.8 |
| Sy.x (Ω.cm^2^) | 5.292 | 638.7 | 162.3 |
| Normality of residuals |  |  |  |
| D’Agostino-Pearson (*p*) | 0.007 | 0.8295 | 0.1414 |
| Anderson-Darling (*p*) | 0.0015 | 0.0388 | 0.1273 |
| Shapiro-Wilk (*p*) | 0.0071 | 0.1733 | 0.2904 |
| Kolmogorov-Smirnov (*p*) | 0.0056 | 0.0096 | > 0.1000 |
| Passed normality tests? | No | Yes | Yes |
| AICc | 129.5 | 628.9 | 376 |
| Sum of Squares | 868.3 | 17539209 | 81732 |
| # of timepoints (X values) | 48 | 48 | 48 |
| # of fitted Y values | 36 | 48 | 36 |

**Figure S3. Results of double sigmoidal modeling of TEER kinetics for MRC-5, hAELVi, and hAELVi/MRC-5 cultures.** This table presents the results of fitting a double sigmoidal model (adapted from Brooks et al. (21)) to TransEpithelial Electrical Resistance (TEER) data collected over 28 days under air-liquid interface (ALI) conditions. The model describes two successive phases of barrier formation, defined by the following parameters: A: maximum TEER value (Ω.cm²), k_1_, k_2_: slopes of the first and second sigmoidal phases (steepness), t_1_, t_2_: time points (in days) at which each transition occurs. Goodness-of-fit was assessed using the adjusted R^2^, the RMSE (root mean square error) representing the average prediction error in Ω.cm^2^, the Sy.x (standard error of the estimate) reflecting residual dispersion, and the AICc (corrected Akaike Information Criterion) penalizing model complexity. Residuals were evaluated for normality using four statistical tests (D’Agostino-Pearson, Anderson-Darling, Shapiro-Wilk, and Kolmogorov-Smirnov). The model showed an excellent fit for hAELVi and hAELVi/MRC-5 cultures, with normally distributed residuals and interpretable kinetics. For MRC-5, the model failed (negative R^2^, poor residual normality), consistent with the absence of epithelial resistance development. Parameters labeled “Unstable” denote poorly constrained estimates with wide or undefined confidence intervals.

*
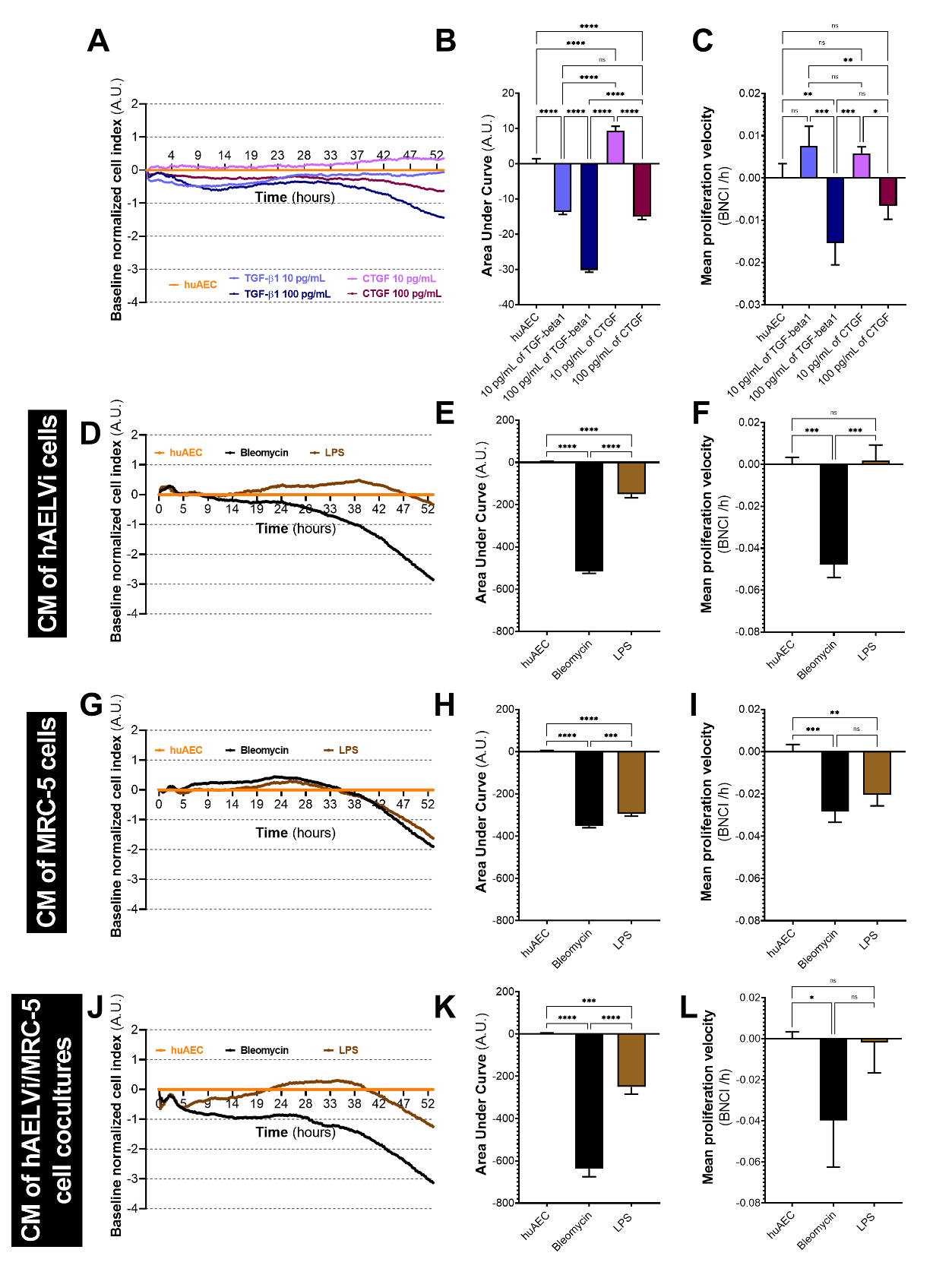
*

**Figure S4. Conditioned media (CM) from lung cells exposed to profibrotic mediators or injurious stimuli impair fibroblast proliferation.** MRC-5 fibroblasts were incubated with either recombinant profibrotic cytokines (A - C) or conditioned media (CM) collected from (D–F) hAELVi monocultures, (G - I) MRC-5 monocultures, or (J - L) hAELVi/MRC-5 co-cultures previously exposed for 24 h to bleomycin (10 µg/mL) or LPS (1 µg/mL). Proliferation was monitored in real time using xCELLigence Real-Time Cell Analysis (RTCA). (A, D, G, J) Time-course of baseline-normalized cell index (BNCI). (B, E, H, K) Area under the BNCI curve. (C, F, I, L) Mean proliferation velocity. Data are shown as mean ± SD of three independent experiments. Statistical analysis was performed by one-way ANOVA followed by Tukey’s multiple comparisons test. *, **, ***, **** indicate *p* < 0.05, 0.01, 0.001, and 0.0001, respectively; ns: not significant.

*
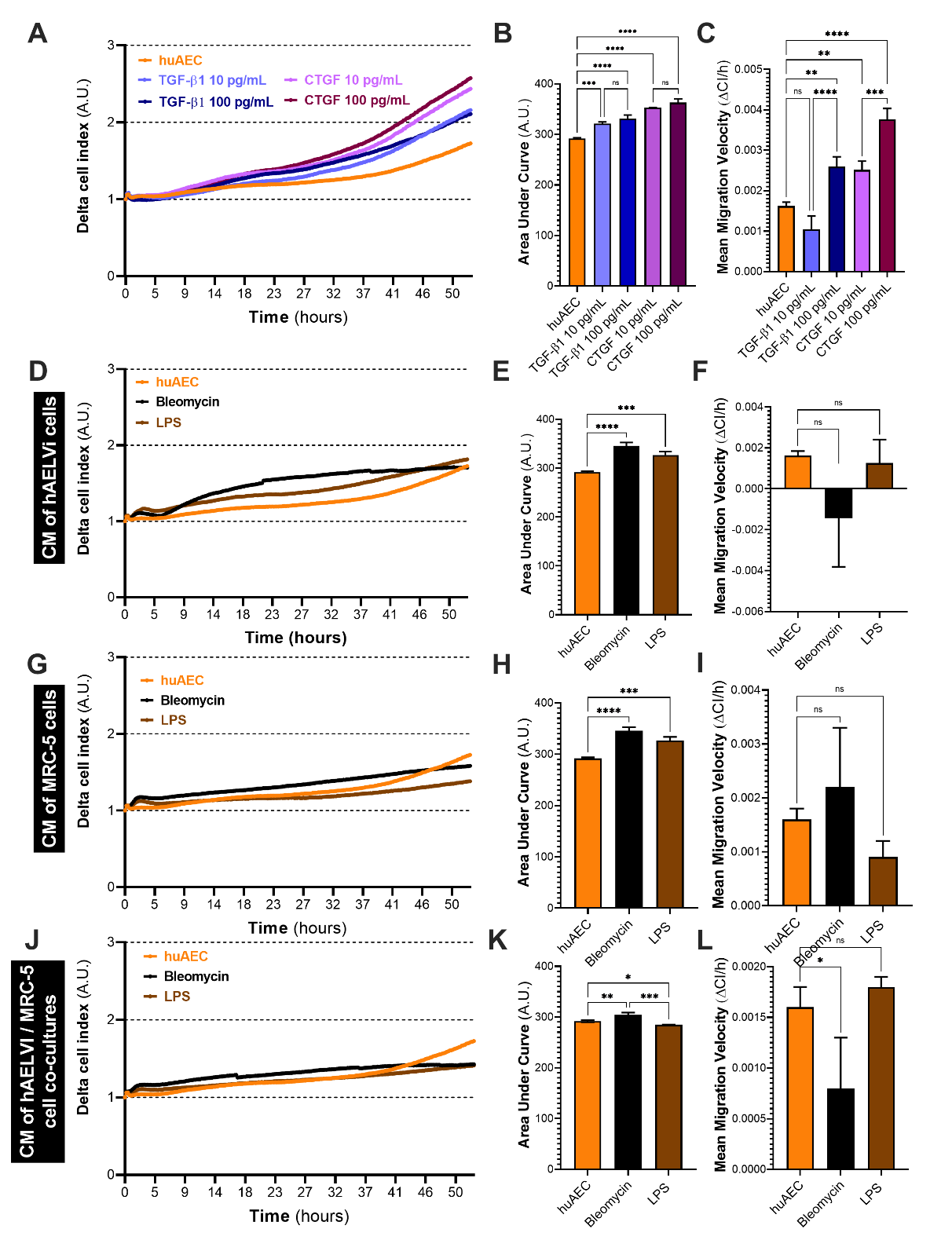
*

**Figure S5. Validation of the fibroblast migration assay and functional response to classical profibrotic or inflammatory stimuli.** MRC-5 fibroblast migration was monitored in real time (xCELLigence system, ΔCI over 53 h) following exposure to (A–C) recombinant profibrotic cytokines or conditioned media (CM) from lung cells previously exposed to bleomycin or LPS under submerged conditions (D-L). Direct stimulation with TGF-β1 or CTGF (10 or 100 pg/mL) in the donor compartment of CIM-plates induced a dose-dependent increase in fibroblast migration (A–C). (A) Migration kinetics (ΔCI), (B) Area Under the Curve (AUC), and (C) Mean migration velocity (ΔCI/h). (D-F) CM from hAELVi cells exposed to bleomycin (0.15 µg/mL) or LPS (1 µg/mL). (G-I) CM from MRC-5 fibroblasts exposed under the same conditions. (J-L) CM from hAELVi/MRC-5 co-cultures exposed in parallel. (D, G, J) Migration kinetics; (E, H, K) AUC quantification; (F, I, L) migration velocity. huAEC (unconditioned medium) was used as baseline control. Data are presented as mean ± SD, n = 4 for huAEC, n = 3 for other conditions. One-way ANOVA followed by Tukey’s post hoc test. **p* < 0.05; ***p* < 0.01; ****p* < 0.001; *****p* < 0.0001; ns: not significant.

**
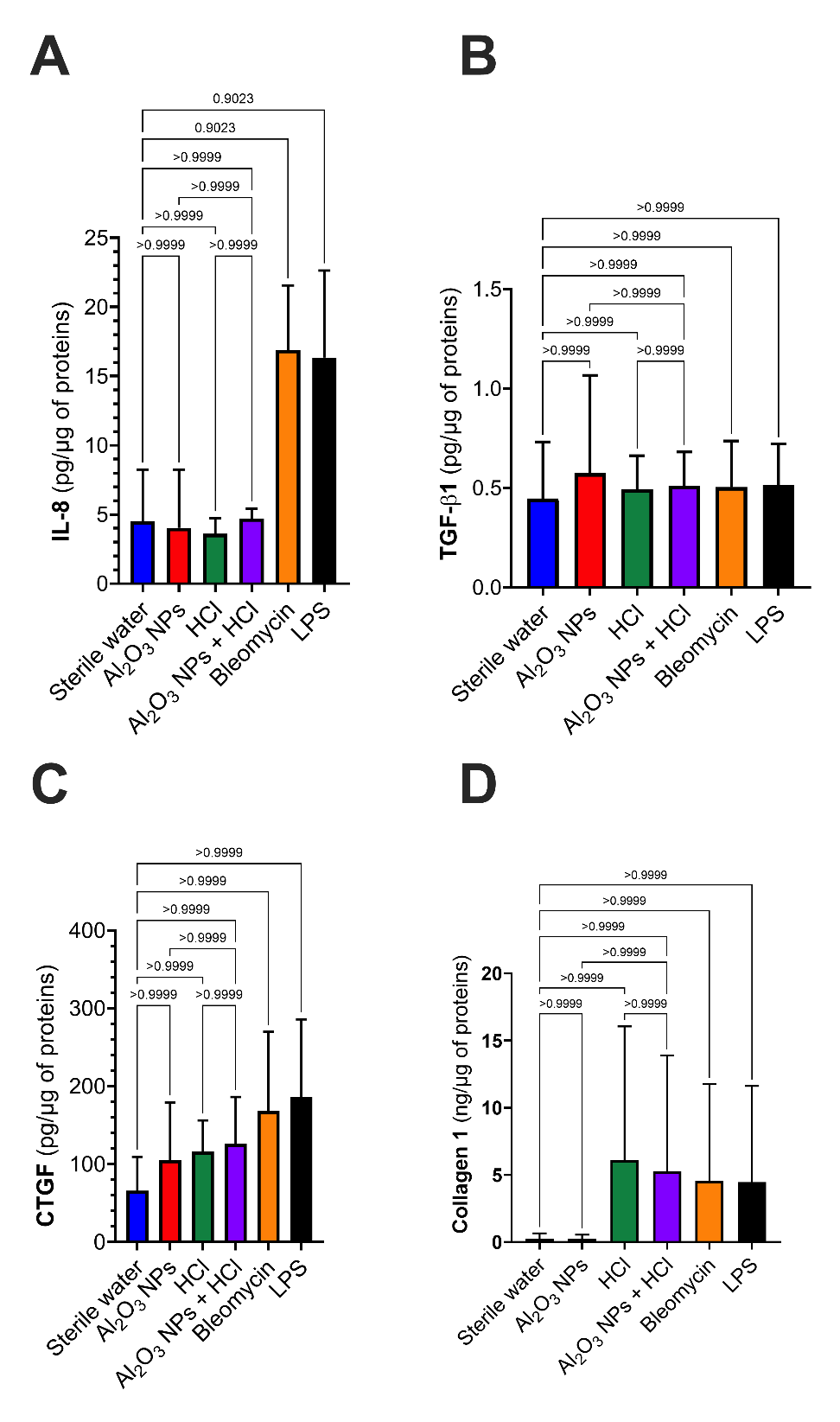
**

**Figure S6. Quantification of secreted proteins by ELISA in hAELVi/MRC-5 co-cultures following ALI exposure.** hAELVi/MRC-5 co-cultures were exposed apically under air-liquid interface (ALI) conditions to sterile water (control), Al₂O₃ nanoparticles (1 mg/mL), HCl (1.37 mM), or their combination using the Vitrocell® CLOUD-12 system. Additional co-cultures were exposed to bleomycin (0.15 µg/mL) or lipopolysaccharide (LPS, 1 µg/mL) as pro-fibrotic and pro-inflammatory positive controls, respectively. Basolateral media were collected 96 hours post-exposure, and levels of IL-8 (A), TGF-β1 (B), CTGF (C), and Collagen I (D) were quantified by ELISA. Results are expressed as mean ± SD (n = 3 independent experiments). Statistical analysis was performed using the Kruskal–Wallis test followed by Dunn’s post hoc test; no significant differences were observed between groups. p-values are shown above each comparison.
